# Characterization and diversity of defense systems in *Providencia* pathogen

**DOI:** 10.3389/fimmu.2026.1755933

**Published:** 2026-02-12

**Authors:** Xiaoyan Li, Yiyan Zhao, Xiang Guo, Yuting Bai, Jinping Wang

**Affiliations:** 1 Department of Clinical Laboratory, Heping Branch, Shanxi Provincial People’s Hospital, Taiyuan, China; 2 Department of Blood Transfusion, Shanxi Provincial People’s Hospital, Taiyuan, China; 3 Shanxi Bethune Hospital, Shanxi Academy of Medical Sciences, Tongji Shanxi Hospital, Third Hospital of Shanxi Medical University, Taiyuan, China; 4 Department of Energy Chemistry and Materials Engineering, Shanxi Institute of Energy, Jinzhong, China; 5 Department of Ultrasound, Beijing Chest Hospital, Capital Medical University & Beijing Tuberculosis and Thoracic Tumor Research Institute, Beijing, China

**Keywords:** antibiotic resistance, CRISPR-Cas, defense systems, Gabija, *Providencia*, Septu, virulence factors

## Abstract

**Introduction:**

*Providencia* species are emerging opportunistic pathogens associated with multidrug-resistant infections, yet their molecular defense mechanisms against phage or mobile genetic elements remain poorly characterized.

**Methods:**

We present a comprehensive pan-genomic analysis of antiviral defense systems across 73 complete genomes (or chromosomes) of *Providencia stuartii* (n = 31) and *Providencia rettgeri* (n = 42), using DefenseFinder and CRISPRCasFinder. We further expanded analysis of contig/scaffold assemblies to confirm conservation of core defense profiles across assembly types. BacMGEnet was employed to derive spacer-MGE interaction networks. Phylogenetic reconstruction and gene gain and loss modeling were performed to assess evolutionary patterns. To validate functionality, we experimentally tested the anti-phage activity of Gabija and Septu in heterologous *E. coli* assays, including point mutation analysis of conserved residues.

**Results:**

We reveal a diverse and complex defense repertoire dominated by restriction-modification systems and CRISPR-Cas Class 1 Type I-F, with significant contributions from toxin-antitoxin, GAPS2, PsyrTA, and Mokosh systems. Notably, defense genes are non-randomly distributed, often clustering into genomic islands suggestive of horizontal acquisition. Expanded analysis confirms conservation of core defense profiles across assembly types, supporting the utility of lower-quality data when complete genomes are scarce. Comparative analysis uncovers species-specific differences, with *P. rettgeri* harboring a higher abundance of non-CRISPR systems. BacMGEnet-derived spacer-MGE interaction networks further highlight species-specific dynamics, dense, hub-driven networks in *P. stuartii* versus sparser networks in *P. rettgeri*. Correlation analysis indicates potential associations between specific defense systems and virulence or antibiotic resistance genes. Phylogenetic reconstruction and gene gain and loss modeling further highlight dynamic evolutionary patterns. Both Gabija and Septu systems conferred robust, phage-specific protection; point mutations in conserved residues (GajA E465K and PtuB H53K) abolished defense.

**Discussion:**

Our findings unveil a multi-layered, modular immune architecture in Providencia, providing crucial insights into its genome plasticity, phage resistance, and adaptation in clinical environments. This work establishes a foundation for understanding the role of defense systems in the evolution and pathogenicity of the *Providencia* genus.

## Introduction

The rise of antimicrobial resistance among bacterial pathogens has emerged as one of the most pressing public health crises, profoundly undermining the efficacy of conventional antibiotics in clinical settings (1). Multidrug-resistant Gram-negative bacteria, in particular, are increasingly implicated in severe nosocomial infections, including bloodstream infections, urinary tract infections, and sepsis, often leading to high morbidity, mortality, and substantial healthcare burdens worldwide (2). In this context, bacteriophage therapy, exploiting natural viruses that specifically infect bacteria, has re-emerged as a promising therapeutic alternative with the potential to circumvent traditional resistance mechanisms (3). However, the success of phage-based interventions is inherently constrained by the extensive and evolving arsenal of prokaryotic immune defenses that bacteria employ to resist mobile genetic elements (MGEs), including bacteriophages and plasmids (4). Far beyond the well-characterized CRISPR-Cas systems, recent genomic studies have uncovered a remarkable diversity of innate defense mechanisms in bacteria and archaea, many of which function analogously to eukaryotic cell-autonomous immunity (5, 6). Understanding the complexity, distribution, and evolutionary dynamics of these defense systems is therefore critical not only for advancing phage therapy but also for elucidating fundamental aspects of microbial survival and pathogenesis.

Over the past decade, our understanding of prokaryotic immune defense systems has undergone a transformative expansion, revealing an unexpectedly rich repertoire of mechanisms that protect bacteria against invasive genetic elements such as bacteriophages and plasmids (5, 7). Traditionally dominated by the study of restriction-modification (RM) systems and CRISPR-Cas adaptive immunity, the field has now uncovered over 100 distinct families of defense systems, collectively referred to as “bacterial innate immunity”, that function through diverse biochemical strategies, including nuclease activation, membrane disruption, abortive infection, and programmed cell dormancy or death (8, 9). Notably, many of these systems exhibit functional parallels to eukaryotic cell-autonomous immunity, such as the use of surveillance proteins that trigger effector responses upon detection of foreign nucleic acids, highlighting deep evolutionary conservation in host defense principles across domains of life (6, 10). The discovery of systems like DISARM, Thoeris, Druantia, and Gabija, among others, underscores the complexity and modularity of bacterial anti-phage defenses, often organized in “defense islands” within microbial genomes (11, 12). Recently, Beavogui et al. introduced the concept of the “defensome” to designate the complete repertoire of bacterial defense systems, which constitutes a pan-immune system (13). Following its introduction, Cunha da Silva and Rossi further explored the defensome of *Actinobacillus pleuropneumoniae*, revealing its complex interplay with MGEs (14). Investigating the pan-immune system is not only essential for understanding bacterial survival, genome stability, and evolutionary dynamics in competitive microbial ecosystems, but also holds profound implications for developing novel antimicrobial strategies (15). A comprehensive characterization of these defense arsenals can inform the rational design of phage cocktails, guide the engineering of phages capable of overcoming host resistance, and uncover new molecular tools for biotechnology and medicine (15).

The genus *Providencia*, belonging to *Proteae* in the *Enterobacteriaceae* family, comprises Gram-negative, facultatively anaerobic, motile bacilli that are widely distributed in diverse environments, including soil, water, and the gastrointestinal tracts of humans and animals (16). As opportunistic pathogens, *Providencia* species, particularly *P. stuartii* (17) and *P. rettgeri* (18), have gained increasing recognition in clinical microbiology due to their association with healthcare-associated infections, especially among immunocompromised individuals, elderly patients, and those with prolonged hospitalization or indwelling medical devices such as urinary catheters (16, 19). Notably, *Providencia* species exhibit intrinsic resistance to multiple antibiotics (20). More alarmingly, they have demonstrated a growing propensity to acquire MGEs harboring extended-spectrum β-lactamases and carbapenemases, leading to drug-resistant phenotypes (21). This escalating antimicrobial resistance, combined with their ability to form biofilms on abiotic surfaces, enhances their persistence in hospital environments and limits therapeutic options, thereby underscoring the urgent need for alternative treatment strategies, including phage therapy (22–24).

Despite the growing clinical importance of *Providencia* pathogens, a comprehensive understanding of their anti-phage defense mechanisms remains largely unexplored. In particular, the diversity, genomic organization, and evolutionary dynamics of defense systems in *P. stuartii* and *P. rettgeri* have not been characterized (25). To address this, we conducted a comparative genomic analysis across the complete genome of *Providencia* isolates, with the primary aim of identifying and classifying the full repertoire of prokaryotic immune systems present in this genus. We also focused on the architecture and diversity of CRISPR-Cas loci and further investigated the co-occurrence and potential interplay between defense systems, virulence factors (VFs), and antibiotic resistance genes (ARGs). Additionally, through phylogeny-aware evolutionary modeling, we reconstructed gene gain and loss events across the *Providencia* phylogenomic tree to assess the selective pressures shaping the expansion and retention of defense-related modules. Our findings reveal the abundance and complexity of defense systems in *Providencia*. This study not only provides an overview of the immune defense landscape in *Providencia* pathogens but also offers crucial insights into their adaptive evolution and survival strategies.

## Results

### Occurrence of defense systems in *Providencia*

To investigate the diversity and distribution of defense systems, we performed a comparative genomic analysis using complete genome sequences of clinically relevant bacterial species associated with sepsis and other severe infections. Genomes were retrieved from the NCBI GenBank database (as of 2025-12-29) and filtered to include only high-quality, closed, and complete genomes (or chromosomes) to ensure accurate identification and annotation of defense systems. A total of 73 *Providencia* strains were analyzed: 31 isolates of *P. stuartii* and 42 isolates of *P. rettgeri*. All accession numbers for the analyzed genomes are listed in **Supplementary Table 1**, enabling reproducibility and further validation of our findings. For comparative purposes, we also included complete genomes from 13 additional sepsis-associated bacterial species across diverse taxonomic groups, including *Clostridioides difficile, Staphylococcus aureus, Yersinia pestis, Mycobacterium tuberculosis*, and *Brucella* spp., among others. This curated dataset allowed us to systematically compare the repertoire of defense systems across closely related and distantly related pathogens, with a particular focus on the exceptional immune complexity observed in *Providencia* species.

**Figure 1:**
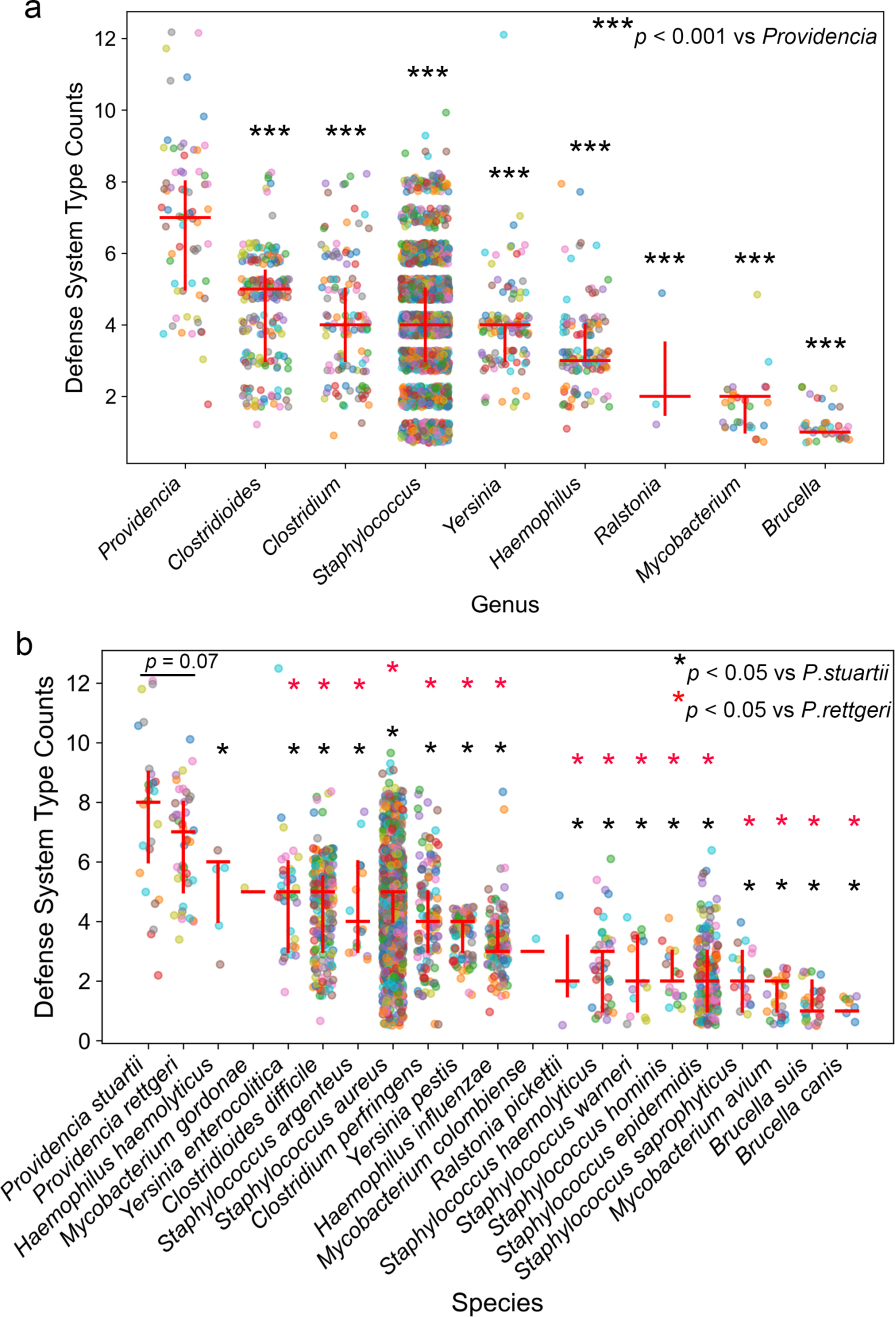
Comparative analysis of immune defense system diversity in *Providencia* and other clinically relevant pathogens. **(A)** Defense system type abundance across bacterial genera associated with sepsis. This panel compares the number of distinct defense system types identified in various bacterial genera known to cause nosocomial infections and sepsis. ***p < 0.001 vs. *Providencia*; one-way ANOVA with Tukey’s *post hoc* test. **(B)** Species-level comparison of defense system richness in *Providencia* and related pathogens. This panel expands the analysis to the species level, focusing on *P. stuartii* and *P. rettgeri*, the two most frequently isolated pathogenic species within the genus, and comparing them with representative species from other sepsis-associated genera. Each colored dot represents an individual genome from a given genus, with jitter applied to reduce overplotting and improve visual resolution. The red crossbars indicate the median (horizontal line) and interquartile range (vertical lines) of defense system counts per genus. The black * (*p* < 0.05) denote significance vs. *P. stuartii*, red * (*p* < 0.05) denote significance vs. *P. rettgeri* (one-way ANOVA with Tukey’s *post hoc* test).

Statistical comparisons of defense system count between groups were performed using one-way ANOVA with Tukey’s *post hoc* test (full details in **Supplementary Data 1**). As illustrated in **Figure 1A**, *Providencia* exhibits one of the highest median counts of distinct defense system types among all sepsis-associated bacterial genera analyzed, with a median of approximately 7 systems per genome, exceeding those observed in *Brucella, Mycobacterium*, and *Ralstonia*, which show median values below 2 (*p* < 0.001; see **Supplementary Data 1** for full pairwise comparisons). Notably, the distribution of defense system richness in *Providencia* is not only elevated but also highly variable, as evidenced by the broad interquartile range (IQR) and the presence of several outliers reaching up to 12 distinct systems. In contrast, other clinically relevant genera such as *Staphylococcus, Clostridium*, and *Yersinia* display more moderate and less variable defense system profiles, with medians ranging from 3 to 6. At the species level (**Figure 1B**), this trend is further reinforced: both *P. stuartii* and *P. rettgeri* consistently harbor a greater number of defense system types compared to most other sepsis-causing species. Remarkably, while *P. rettgeri* shows a slightly higher median count than *P. stuartii*, the two species are nearly indistinguishable in the total number of defense system per genome (*p* = 0.07), with both exhibiting substantial intra-species variation and frequent occurrences of genomes carrying more than 8 different defense systems. Statistical comparisons reveal significant differences between *P. stuartii* and *P. rettgeri* and most other species (*p* < 0.05), with black asterisks indicating significance relative to *P. stuartii* and red asterisks indicating significance relative to *P. rettgeri*.

To further resolve the architectural complexity of these immune systems, we analyzed the abundance of defense system subtypes, specific molecular variants within each system class (e.g., CRISPR-Cas type I-E, II-A; R-M Type I, II, etc.), across the same set of genomes. As shown in **Supplementary Figure 1**, *Providencia* again stands out with the highest median subtype count (~8 subtypes per genome), surpassing all other genera (*p* < 0.001). At the species level (**Supplementary Figure 1B**), *P. stuartii* and *P. rettgeri* exhibit higher subtype richness compared to most other clinical pathogens, with some strains harboring over 10 distinct subtypes. Significant differences were observed between *P. stuartii* (or *P. rettgeri*) and most other species (*p* < 0.05), marked by black and red asterisks, respectively; full statistical details are available in **Supplementary Data 1**. This elevated subtype diversity indicates not only a greater number of defense systems but also a broader functional repertoire, suggesting that *Providencia* may deploy a multi-layered, modular defense strategy against invading genetic elements.

### Characterization of defense systems in *Providencia*

**Figure 2:**
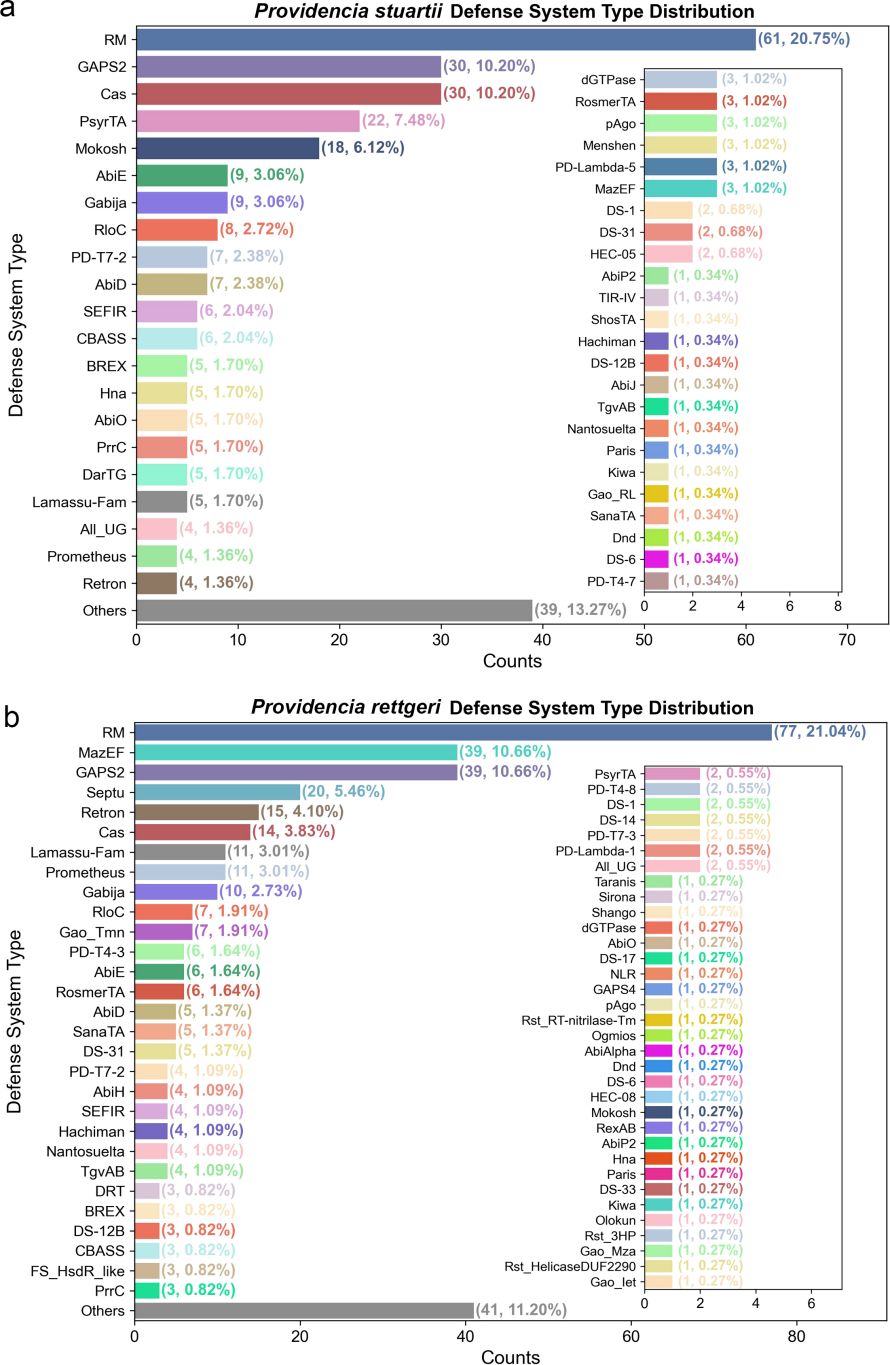
Composition and distribution of immune defense systems in *P. stuartii* and *P. rettgeri*. **(A)** Defense system type distribution in *P. stuartii*. Bar plot showing counts (and percentages) of defense system types in *P. stuartii* complete genomes (n = 31). The main panel displays dominant systems, while the inset plot details the “Others” category (13.27% of systems). **(B)** Defense system type distribution in *P. rettgeri*. Bar plot showing counts (and percentages) of defense system types in *P. rettgeri* complete genomes (n = 42). The main panel displays dominant systems, while the inset plot details the “Others” category (11.20% of systems).

To gain deeper insight into the composition and prevalence of defense systems in *Providencia*, we performed a detailed profiling of immune mechanisms across all analyzed genomes of *P. stuartii* (n = 31) and *P. rettgeri* (n = 42). As shown in **Figures 2A, B**, the most abundant defense system type in both species is the restriction-modification (RM) system, with 61 occurrences (20.75%) in *P. stuartii* and 77 occurrences (21.04%) in *P. rettgeri*, respectively, highlighting its fundamental role in innate immunity against foreign DNA. The CRISPR-Cas system is among the most abundant defense systems in both species, detected in 30 strains (10.20%) in *P. stuartii* and 14 strains (3.83%) in *P. rettgeri*, representing 10.73% and 11.99% of total defense systems, respectively. Other systems include GAPS2 (30, 10.20% in *P. stuartii*; 39, 10.66% in *P. rettgeri*) and Gabija (9, 3.06% in *P. stuartii*; 10, 2.73% in *P. rettgeri*), underscoring the presence of multiple non-CRISPR defense mechanisms. Notably, while RM and CRISPR-Cas systems are prevalent in both species, *P. rettgeri* exhibits higher counts of several non-CRISPR systems such as MazEF (39, 10.66%), Retron (15, 4.10%), and Lamassu-Fam (11, 3.01%), suggesting potential differences in evolutionary adaptation or niche-specific immune pressures. In contrast, *P. stuartii* shows greater representation of PsyrTA, Mokosh (18, 6.12%), and AbiE (9, 3.06%), indicating distinct immune prioritization between the two species. The full spectrum of defense systems is shown in the main panels (**Figures 2A, B**), with inset plots detailing the composition of the “Others” category, which accounts for 13.27% in *P. stuartii* and 11.20% in *P. rettgeri*. This comprehensive view confirms the presence of numerous rare but functionally distinct systems, including ShosTA, Thoeris, pAgo, and Kiwa, with some occurring in only one or two strains, emphasizing the high degree of genomic heterogeneity and modular evolution within the genus. All raw counts and system classifications are provided in **Supplementary Data 2**. Together, these findings illustrate a complex, multi-layered defense architecture in *Providencia*, dominated by RM and CRISPR-Cas systems but enriched by a wide array of auxiliary immune mechanisms that likely contribute to phage resistance and genome stability in diverse environments.

To expand the scope of our defense system profiling beyond complete genomes, we analyzed *P. stuartii* and *P. rettgeri* using additional genomic assemblies at the contig and scaffold levels, which are more widely available in public databases like NCBI. Specifically, we applied DefenseFinder to 429 contigs and 50 scaffolds of *P. stuartii*, and 334 contigs and 244 scaffolds of *P. rettgeri*. As shown in **Supplementary Figures 2A–D**, this broader dataset reveals a significantly higher total number of defense system occurrences due to increased genomic sampling. In *P. stuartii*, the most abundant systems across all contigs are RM (608, 15.31%) and Cas (429, 10.80%), followed by GAPS2 (425, 10.70%) and PsyrTA (409, 10.30%). Similarly, in *P. rettgeri*, RM (567, 18.50%) and GAPS2 (326, 10.64%) dominate, with MazEF (322, 10.51%) and Gabija (128, 4.18%) also highly represented. Notably, the relative abundance of major systems such as RM, Cas, GAPS2, and PsyrTA remains consistent across complete, contig and scaffold datasets, suggesting robust detection of prevalent defense mechanisms even in fragmented assemblies. While full-length genome assemblies are ideal for accurate defense system annotation, since correct gene order, operon structure, and strand orientation are critical for functional inference, the results from contig and scaffold data demonstrate that they can still capture dominant immune features. This suggests that such fragmented datasets may serve as useful proxies for preliminary assessments of defense system prevalence, particularly when complete genomes are scarce.

**Figure 3:**
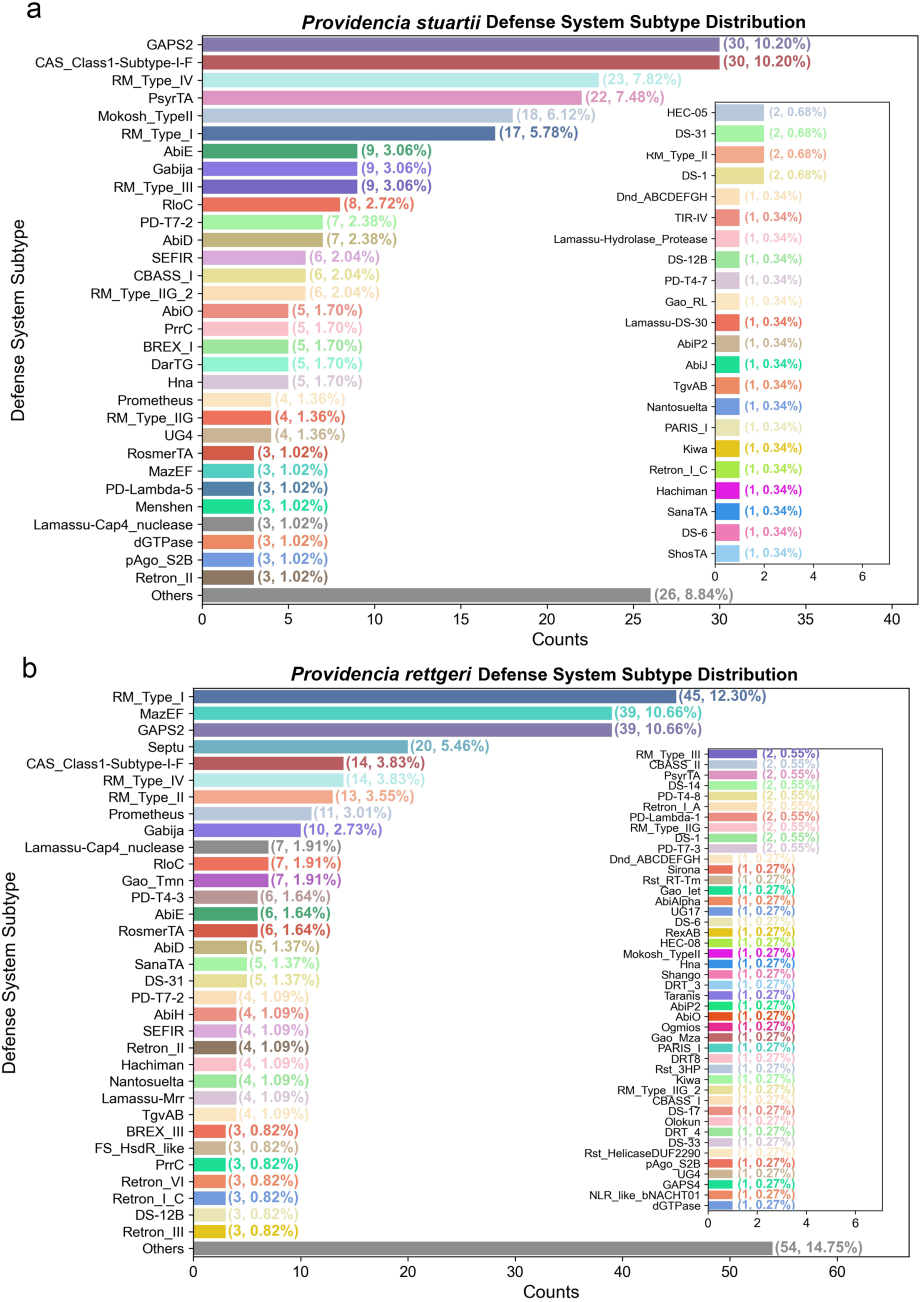
Composition and distribution of immune defense system subtypes in *P. stuartii* and *P. rettgeri*. **(A)** *P. stuartii* defense system subtype distribution. Bar plot showing counts (and percentages) of defense system subtypes in *P. stuartii* complete genomes (analyzed via DefenseFinder). Dominant subtypes include *RM_Type-IV* (23, 7.82%) and *CAS_Class1-Subtype-I-F* (30, 10.20%); the inset plot details the “Others” category (8.84% of subtypes). **(B)** *P. rettgeri* defense system subtype distribution. Bar plot showing counts (and percentages) of defense system subtypes in *P. rettgeri* complete genomes (analyzed via DefenseFinder). Dominant subtypes include *RM_Type-I* (45, 12.30%) and *MazEF* (39, 10.66%); the inset plot details the “Others” category (14.75% of subtypes).

We next analyzed the distribution of defense system subtypes, offering a higher-resolution view of functional diversity. As shown in **Figures 3A, B**, the most abundant subtype across both species is RM subtypes, exhibit substantial diversity, with RM_Type_I, RM_Type_II, RM_Type_IV, and et al. collectively accounting for more than 20% of all subtypes, reflecting a multi-layered DNA surveillance mechanism. Notably, the CRISPR-Cas system is represented almost exclusively by a single subtype: CAS_Class1-Subtype-I-F, across both species, with 30 occurrences (10.22%) in *P. stuartii* and 14 (3.83%) in *P. rettgeri*. This subtype dominates the CRISPR-Cas landscape, and no other known CRISPR subtypes such as I-E, II-A, or III-B, were detected in either species, indicating a highly focused evolutionary strategy for adaptive immunity in *Providencia*. Notably, the “Others” category accounts for 26 (8.84%) in *P. stuartii* and 54 (14.75%) in *P. rettgeri*, encompassing rare but functionally distinct variants such as PARIS_I, Thoeris_II, and DS-1, many of which appear in only one or two isolates. The full list of subtypes and their counts is provided in **Supplementary Data 2**. Together, these findings highlight a unique immune profile in *Providencia*: dominated by a narrow set of highly prevalent subtypes, yet enriched with a diverse array of auxiliary systems.

To assess the consistency and comprehensiveness of subtype annotation, we performed an independent analysis using the PADLOC tool on the same set of complete genomes, complementing our prior DefenseFinder results. As shown in **Supplementary Figures 3A, B**, PADLOC identifies a broadly similar profile of dominant defense systems, with RM types collectively being the most abundant subtype in both species. And cas_type_I-F1 also highly represented, confirming the robust detection of these major immune modules across platforms. This concordance supports the reliability of the core defense repertoire observed in our initial analysis. However, PADLOC also reveals several previously undetected or underrepresented systems, particularly within the DMS_other and PDC family, which are not annotated by DefenseFinder. While both tools agree on the dominance of RM and CRISPR-Cas systems, PADLOC’s sensitivity to divergent or atypical architectures allows it to detect additional immune variants, highlighting the importance of multi-tool validation in defense system profiling. However, it should be noted that the PADLOC database has not been updated since its last release, and thus lacks recently characterized systems. Unlike DefenseFinder, which directly annotates both system types and subtypes, PADLOC outputs predictions exclusively at the subtype level, necessitating post grouping to reconstruct higher-order system categories.

**Figure 4:**
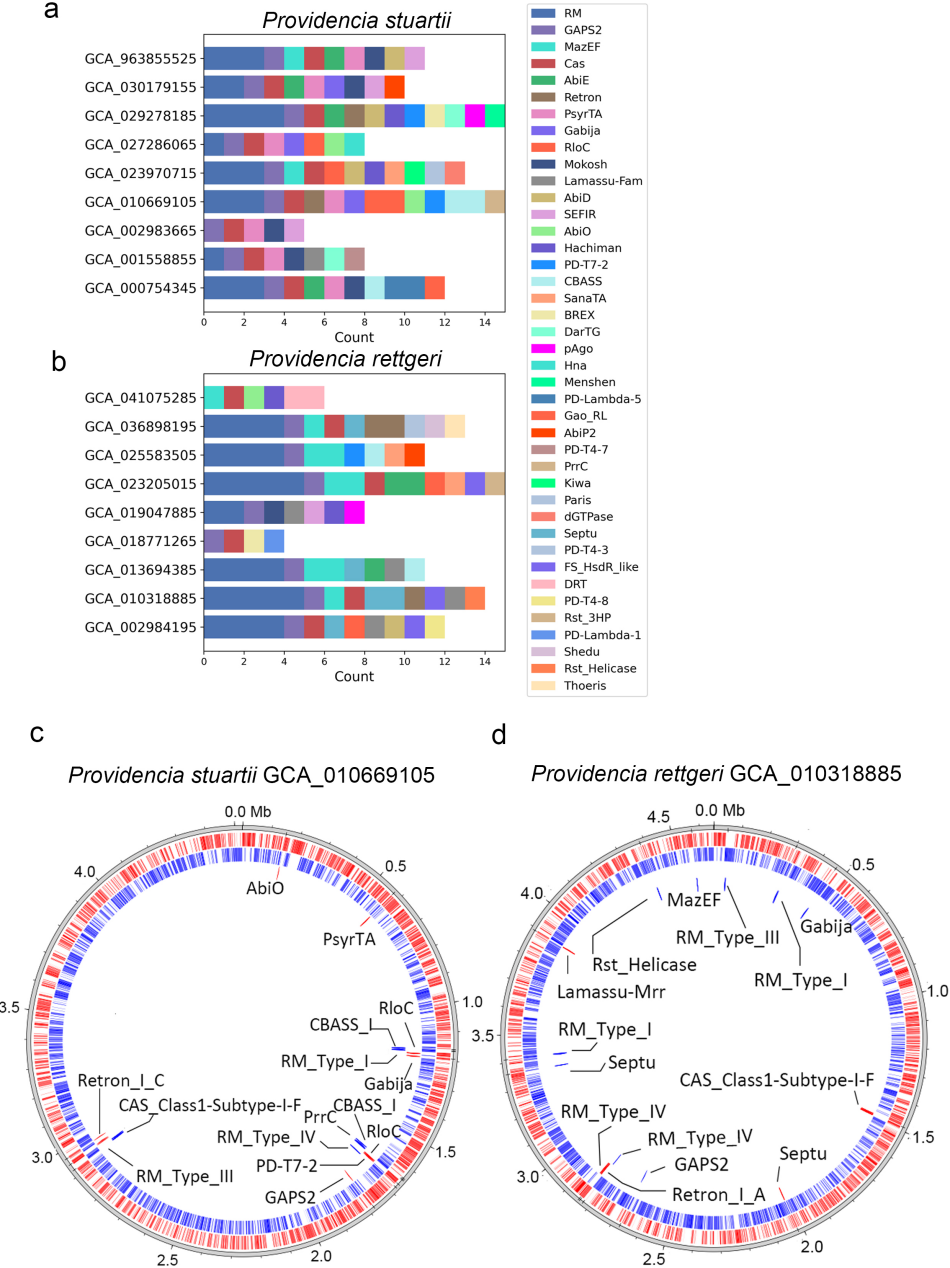
Genomic distribution and composition of defense system in selected *P. stuartii* and *P. rettgeri* strains. It illustrates the diversity and genomic organization of defense system subtypes across selected *P. stuartii* **(a, c)** and *P. rettgeri* **(B, D)** strains. **(A, B)** Present stacked bar charts showing the number of distinct defense system (subtypes) present in individual isolates, with each color representing a specific subtype as defined in the legend. The horizontal axis indicates the count of each system per strain, revealing substantial inter-strain variation in immune repertoire composition. **(C, D)** Display circular genome maps of representative strains, *P. stuartii* GCA_010669105 and *P. rettgeri* GCA_010318885, annotating all identified defense system at their genomic locations. Red and blue indicating genes transcribed in opposite directions of circular genome; typically, functional defense operons exhibit co-directional gene arrangement, suggesting coordinated expression.

To examine the inter-strain variation in immune defense systems, we analyzed the genomic composition and organization of defense system types across a representative subset of *P. stuartii* and *P. rettgeri* isolates. **Figures 4A, B** show stacked bar charts depicting the number and types of defense system subtypes present in individual strains, revealing substantial heterogeneity in immune repertoire composition. For example, *P. stuartii* strain GCA_010669105 harbors 15 distinct subtypes, while other strains contain as few as 5, indicating significant variability in immune complexity even within the same species. Similarly, *P. rettgeri* strains exhibit diverse profiles, with some carrying up to 14 subtypes (e.g., GCA_0103188815), while others possess fewer than 4. Stacked bar charts illustrating all analyzed strains can be found in **Supplementary Figure 4A** (*P. stuartii*) and **4B** (*P. rettgeri*). The circular genome maps in **Figures 4C, D** reveal the distribution of defense systems in the genomes of two strains. In *P. stuartii* GCA_010669105, three major defense-associated genomic islands (GIs) are evident: one near 1.2 Mb; another at ~1.7 Mb; and a third near 3.0 Mb. These clusters are characterized by co-directional gene transcription (indicated by red/blue arrows), consistent with operon-like organization. In contrast, most defense systems appear as isolated, singleton loci in *P. rettgeri* GCA_010318885; seems to be more randomly located in the genome. The presence of structured defense islands and dispersed systems may suggest a dual evolutionary strategy: one favoring stable, co-adapted gene clusters for robust immunity, and another allowing flexible, piecemeal adaptation to novel threats.

### Diversity of CRISPR-Cas systems in *Providencia*

**Figure 5:**
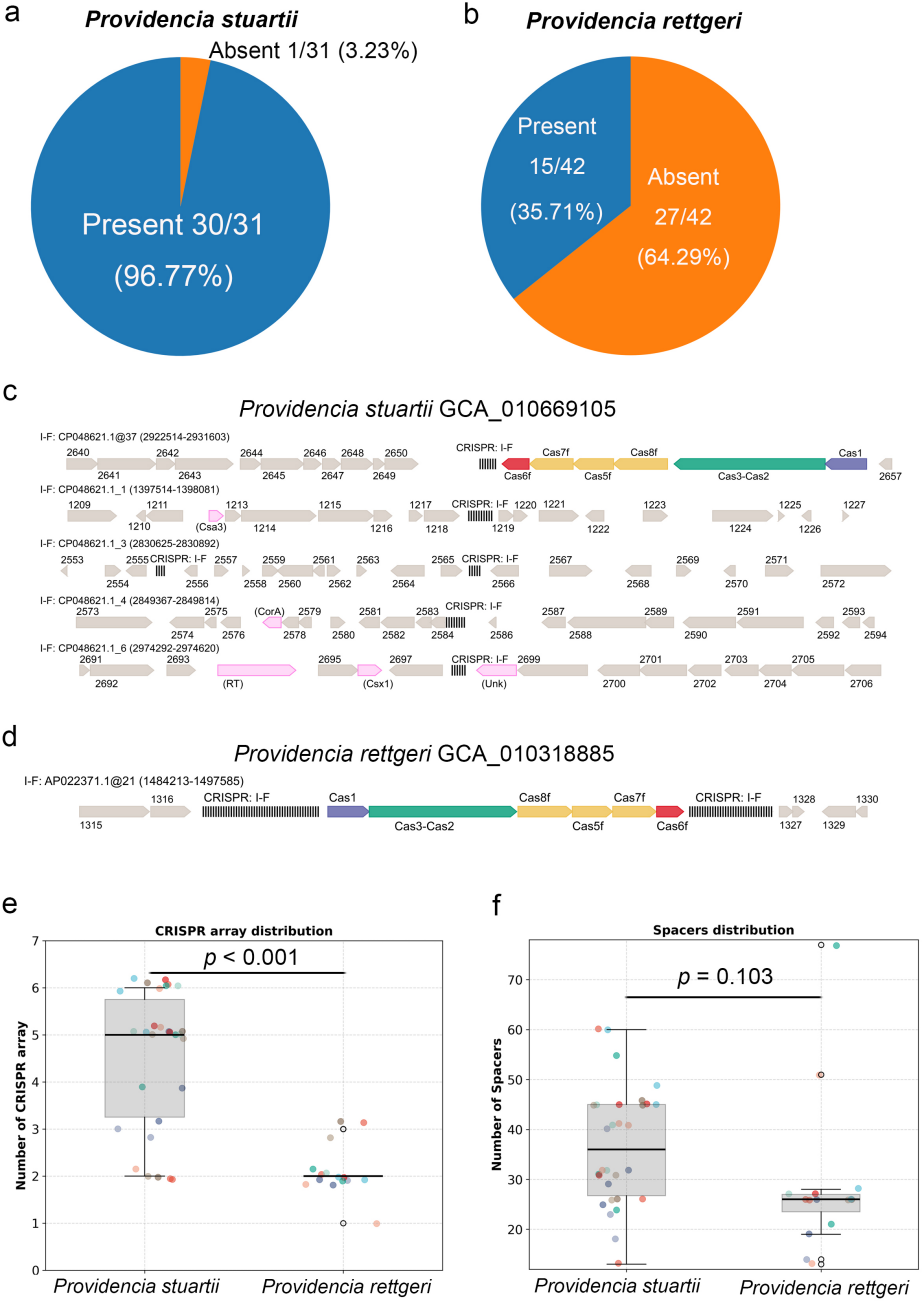
Comparative analysis of CRISPR-Cas systems across *P. stuartii* and *P. rettgeri*. **(A, B)** Show pie charts depicting the proportion of strains harboring at least one CRISPR-Cas system, in *P. stuartii*. **(C, D)** Illustrate representative CRISPR-Cas loci from each species. In both cases, the systems are predominantly of Class 1, Subtype I-F, with conserved *cas* gene arrays, depicted as colored arrows proportional to their genomic length and orientation. The black vertical lines represent individual spacers within the repeat-spacer arrays, which encode sequence-specific immunity against phages and plasmids, contributing to adaptive defense diversity. **(E)** Presents a boxplot showing the distribution of CRISPR array counts across individual strains, with each colored point representing one genome and jitter applied to improve visualization. **(F)** displays a boxplot of spacer counts across all CRISPR-positive strains, with each colored point representing an individual strain and jitter applied for clarity. Each colored circle represents a single strain genome, while outliers are marked with open circles.

The CRISPR-Cas system is a key component of adaptive immunity in bacteria, and its distribution across *Providencia* species reveals a striking pattern of both conservation and divergence. As shown in **Figures 5A, B**, the presence of CRISPR-Cas systems varies significantly between *P. stuartii* and *P. rettgeri*. In *P. stuartii*, the system is nearly ubiquitous, with 30 out of 31 strains (96.77%) harboring at least one CRISPR array, whereas in *P. rettgeri*, only 15 out of 42 strains (35.71%) possess this defense mechanism, indicating a substantial difference in the prevalence of CRISPR-based immunity between the two species. Actually, this contrast is supported by the subtype-level analysis in **Figure 3** and **Supplementary Figures 3A, B**, which shows that CAS_Class1-Subtype-I-F is the sole CRISPR-Cas subtype detected across all analyzed genomes (All data are provided in **Supplementary Data 3**). The exclusive dominance of this single subtype suggests a highly specialized evolutionary trajectory for adaptive immunity in *Providencia*. Notably, while both species carry the same subtype, its relative abundance differs markedly, indicating not only differential acquisition but also varying degrees of functional integration into the immune repertoire. These findings collectively highlight a unique CRISPR landscape in *Providencia*: characterized by a narrow specificity of Cas subtype and a pronounced species-level disparity in prevalence, suggesting distinct evolutionary strategies for phage defense.

At the genomic level, the architecture of the CRISPR system further distinguishes *P. stuartii* from *P. rettgeri*, despite both species typically encoding only a single copy of the core Cas operon. As illustrated in **Figures 5C, D**, the Cas protein cluster, comprising *cas1*, *cas3*, and the other *cas* genes, is highly conserved in gene content and order across both species, consistent with the canonical Class 1 Type I-F system. However, the organization of CRISPR arrays relative to this Cas locus is different. In *P. stuartii*, multiple CRISPR arrays are often found dispersed across the chromosome, with some located near the Cas operon and others situated at distant genomic loci, up to several hundred kilobases away. This arrangement suggests that a single Cas complex may function with multiple spatially separated arrays,. In contrast, *P. rettgeri* strains exhibit a much more restricted configuration: CRISPR arrays are almost exclusively located immediately upstream or downstream of the Cas operon, forming a compact, self-contained unit. This localized architecture implies a more limited and tightly coupled system, where the Cas machinery acts primarily on a single or few adjacent arrays. The distinct genomic distribution of CRISPR repeats thus reinforces the functional divergence between the two species, not only is the CAS_Class1-I-F system more prevalent in *P. stuartii*, but its genomic integration also supports a more expansive and potentially flexible immune strategy.

The number and distribution of CRISPR arrays not only reflect the complexity of the immune system but may also influence spacer acquisition efficiency and system stability. While the structure of typical CRISPR arrays has been illustrated, whether all CRISPR arrays follow the same trend in terms of their distribution and quantity. To further evaluate the architectural diversity, we analyzed the number of CRISPR arrays per strain across all CRISPR-positive isolates (**Figure 5E**). The boxplot reveals a difference between *P. stuartii* and *P. rettgeri*: while *P. stuartii* strains harbor 2 to 6 CRISPR arrays, with a median of 5 arrays per genome, *P. rettgeri* strains are predominantly limited to 2 arrays, with only a few exceptions reaching up to 3. Importantly, this multi-array architecture aligns with the dispersed genomic organization seen in **Figure 5C**, where multiple CRISPR repeats are located at distant chromosomal positions. In contrast, the limited number of arrays in *P. rettgeri*, often clustered near the Cas operon, reflects a more streamlined and possibly less flexible immune configuration. Together, these results indicate that the expansion of CRISPR arrays is an important determinant of adaptive immunity capacity, and that *P. stuartii* has evolved a more complex and scalable system compared to *P. rettgeri*, reinforcing the notion of species-specific evolutionary trajectories in CRISPR-mediated defense.

Given that differences already exist between *P. stuartii* and *P. rettgeri* in terms of CRISPR system distribution and genomic structure, do these two species exhibit further differentiation at the functional level, specifically regarding the number and diversity of spacers. To assess the functional potential, we analyzed the number of spacers per strain across all CRISPR-positive isolates (**Figure 5F**). The median spacer count in *P. stuartii* is much higher, approximately 35 spacers per strain, with a broad interquartile range and multiple strains exceeding 60 spacers, indicating not only a greater capacity to record past infections but also a more extensive immunological memory. In contrast, *P. rettgeri* strains exhibit a substantially lower median of ~25 spacers, with most strains clustering between 20 and 30, and only one outlier surpassing 70. Furthermore, the distribution of spacer counts in *P. stuartii* is more heterogeneous, whereas *P. rettgeri* displays a narrower and more uniform profile. This disparity suggests that *P. stuartii* not only maintains CRISPR-Cas systems at a higher frequency (**Figure 5A**) and with greater genomic flexibility (**Figure 5C**), but also accumulates spacers at an elevated rate, likely due to more frequent exposure to phages or more efficient spacer acquisition mechanisms.

**Figure 6:**
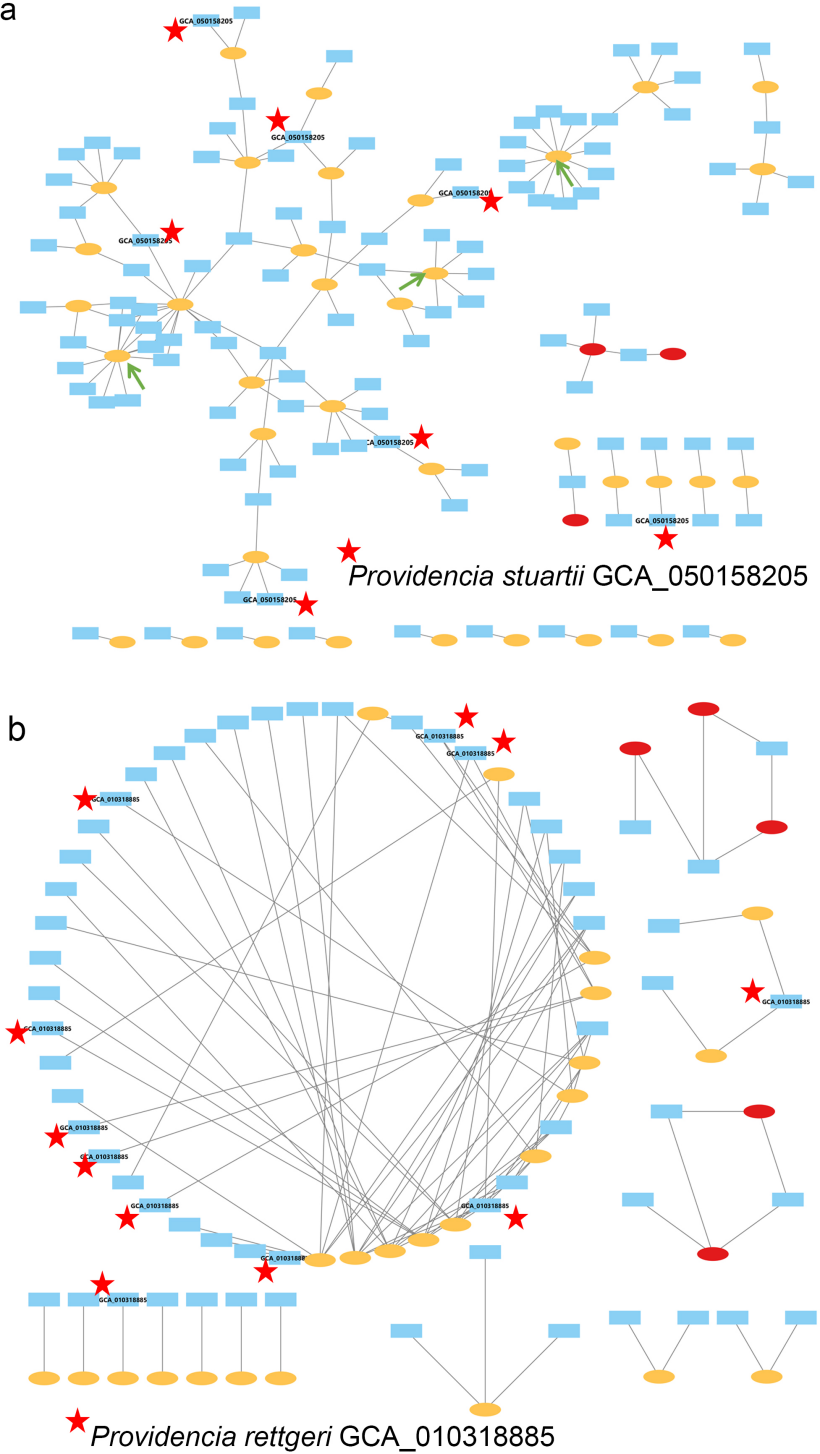
Spacer-mobile genetic element (MGE) interaction networks of *P. stuartii* and *P. rettgeri.* **(a)** Spacer-MGE interaction network of *P. stuartii*. Blue nodes represent CRISPR spacers, with red stars indicating unique spacers derived from the representative strain (GCA_050158205). Yellow ovals denote phages, and red ovals represent plasmids. An edge connects a spacer and an MGE if the spacer sequence matches a protospacer in the MGE (filter criteria: >90% sequence identity, >80% query coverage, e-value <0.001). **(b)** Spacer-MGE interaction network of *P. rettgeri*. Nodes and edges follow the same color and definition conventions as **(a)**, with red stars marking spacers from the representative strain (GCA_010318885). All networks were constructed using the BacMGEnet pipeline, with non-redundant spacers and MGEs selected via a greedy algorithm, and visualized in Cytoscape.

CRISPR spacers serve as molecular memory of past encounters between bacteria and MGEs, with each spacer derived from a protospacer sequence in an invading phage or plasmid. By matching these spacers to known MGE databases (the BacMGEnet pipeline), we reconstructed spacer-MGE interaction networks for *P. stuartii* and *P. rettgeri*. In total, 546 unique spacers from *P. stuartii* and 363 from *P. rettgeri* were identified, of which 110 and 61 matched phages or plasmids, respectively. As shown in **Figures 6A, B**, the resulting networks reveal distinct patterns of spacer-MGE interactions. The green arrows in the *P. stuartii* spacer-MGE interaction network highlight two distinct clusters where multiple spacers from different strains converge on a single phage (yellow oval), indicating shared immunity against common viral threats. These highly connected hubs suggest that certain phages have repeatedly infected multiple *P. stuartii* strains, leading to the acquisition of identical or highly similar spacers. In contrast, the *P. rettgeri* network lacks such densely interconnected MGE nodes, with most phages linked to limited strains. These findings suggest that CRISPR-based immunity plays a key role in shaping bacterial population dynamics.

In addition, spacers enable the reconstruction of host-MGE interaction networks by linking bacterial strains to the phages or plasmids they have encountered. In our analysis, the *P. stuartii* network comprises 56 nodes: 18 host strains and 38 MGEs (37 phages and 1 plasmid); while the *P. rettgeri* network includes 40 nodes: 13 hosts and 27 MGEs (24 phages and 3 plasmids) (**Supplementary Figures 5A, B**). The *P. stuartii* network displays a highly interconnected architecture with numerous edges, indicating frequent and shared targeting of phages across multiple strains. By contrast, the *P. rettgeri* network is markedly sparser, with fewer connections and more isolated host-MGE pairs, consistent with its lower CRISPR array abundance and spacer diversity observed earlier. Notably, several *P. stuartii* strains interact with multiple phages, and certain phages are targeted by multiple hosts, suggesting the emergence of community-level immunity. Together, these distinctions underscore the value of network-based approaches in uncovering ecological and evolutionary dynamics of host-phage interactions and provide a foundation for future investigations into phage resistance mechanisms and the potential for precision phage therapy in *Providencia* infections.

Furthermore, the sequence characteristics, diversity, and structural stability of CRISPR repeats are not only the foundation of CRISPR system function but may also influence crRNA processing efficiency, Cas protein recognition ability, and overall immune activity. Analysis of repeat characteristics across *Providencia* genomes revealed diversity within both species (**Table 1**). A total of 19 distinct repeat variants were identified, indicating that both species maintain multiple repeat types, potentially supporting functional heterogeneity among CRISPR loci. Notably, the number of spacers associated with each repeat type varies dramatically, from as few as 4 to over 300, reflecting differential expansion dynamics and highlighting that certain repeat loci are hotspots for spacer acquisition and immune memory accumulation. The predicted RNA secondary structures of representative repeats (**Supplementary Figures 6**, **7**) further reveal their molecular architecture. Both *P. stuartii* and *P. rettgeri* repeats form stable stem-loop structures, consistent with the canonical hairpin required for Cas protein recognition and crRNA maturation.

**Table 1:** The characteristics of repeats in CRISPR array of *Providencia*.

Species	Consensus	Length	Spacer^a^	MFE^b^ (kcal/mol)
*Providencia rettgeri*	GTTCACCGCCACACAGGCGGCTTAGAAA	28	39	-9.41
	GTTCACCGCCATACAGGCGGCTTAGAAA	28	4	-9.30
	GTTCACTGCCATACAGGCAGCTTAGAAA	28	30	-7.80
	GTTCACTGCCATGCAGGCAGCTTAGAAA	28	32	-7.80
	GTTCACTGCCGTATAGGCAGCTTAGAAA	28	117	-8.60
	TTTCTAAGCCGCCTGTATGGCGGTGAAC	28	13	-11.30
	TTTCTAAGCCGCCTGTGTGGCGGTGAAC	28	20	-11.30
	TTTCTAAGCCGCCTGTGTGGCGGTGCAC	28	27	-11.30
	TTTCTAAGCTGCCTATACGGCAGTGAAC	28	148	-9.10
	TTTCTAAGCTGCCTGTATGGCAGTGAAC	28	16	-9.80
*Providencia stuartii*	GGTGTACTGCCGTATAGGCAGCTTA	25	4	-8.60
	GTGTACTGCCGCATAGGCAGCTTAGAAA	28	83	-8.60
	GTGTACTGCCGTATAGGCAGCTTAGAAA	28	66	-8.60
	GTGTACTGCCGTATAGGCAGCTTAGAAAA	29	9	-8.60
	GTTCACTGCCGCATAGGCAGCTTAGAAA	28	73	-8.60
	GTTCACTGCCGTATAGGCAGCTTAGAAA	28	314	-8.60
	TTTCTAAGCTGCCTATACGGCAGTACAC	28	90	-9.60
	TTTCTAAGCTGCCTATACGGCAGTGAAC	28	337	-9.10
	TTTCTAAGCTGCCTATACGGCAGTGCACATAA	32	4	-9.10
	TTTCTAAGCTGCCTATGCGGCAGTAAAC	28	8	-9.60
	TTTCTAAGCTGCCTATGCGGCAGTACAC	28	52	-9.60
	TTTCTAAGCTGCCTATGCGGCAGTGAAC	28	69	-9.10

MFE, minimum free energy.

a The number of spacers separated by the corresponding consensus repeats.

b The minimum free energy (MFE) corresponding to the optimal secondary structure of the corresponding consensus repeats.

### Correlation of defense systems and ARGs or VFs

In addition to immune defense systems, the pathogenic potential of *Providencia* species is critically shaped by two key genomic determinants: antibiotic ARGs and VFs. ARGs enable these bacteria to survive under antimicrobial pressure, posing significant challenges in clinical settings where treatment options may be limited. Meanwhile, VFs, such as adhesins, toxins, secretion systems, and iron acquisition systems, facilitate host colonization, tissue invasion, and immune evasion, thereby driving infection progression. The co-occurrence of defense systems with ARGs and VFs within the same genomic contexts can promote the coordinated dissemination of multiple adaptive traits through HGT, potentially giving rise to multidrug-resistant, highly virulent clones. Therefore, understanding the prevalence, diversity, and genomic localization of ARGs and VFs is essential for comprehensively characterizing the evolutionary and clinical significance of *Providencia* pathogens.

**Figure 7:**
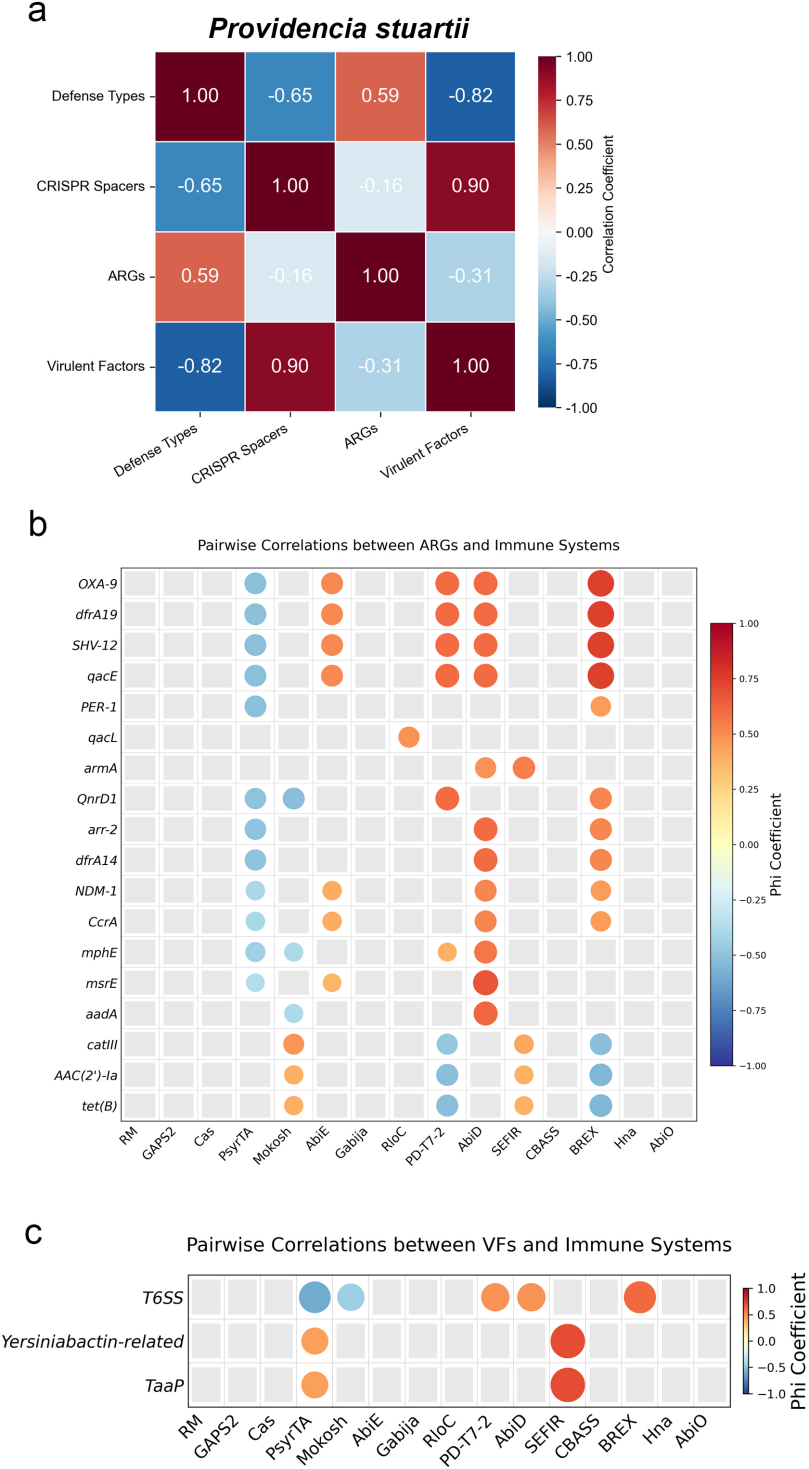
Correlation analysis between defense systems and antimicrobial resistance genes (ARGs) or virulence factors (VFs) in *P. stuartii*. **(A)** Correlation heatmap showing pairwise relationships among key variables: total number of defense system types, CRISPR spacer count, ARG count, and VF count. **(B, C)** Display pairwise correlation matrices using the Phi coefficient, with circle size and color intensity reflecting the strength and direction of association. Only values with a *p*-value less than 0.1 are displayed in the figure.

**Figure 8:**
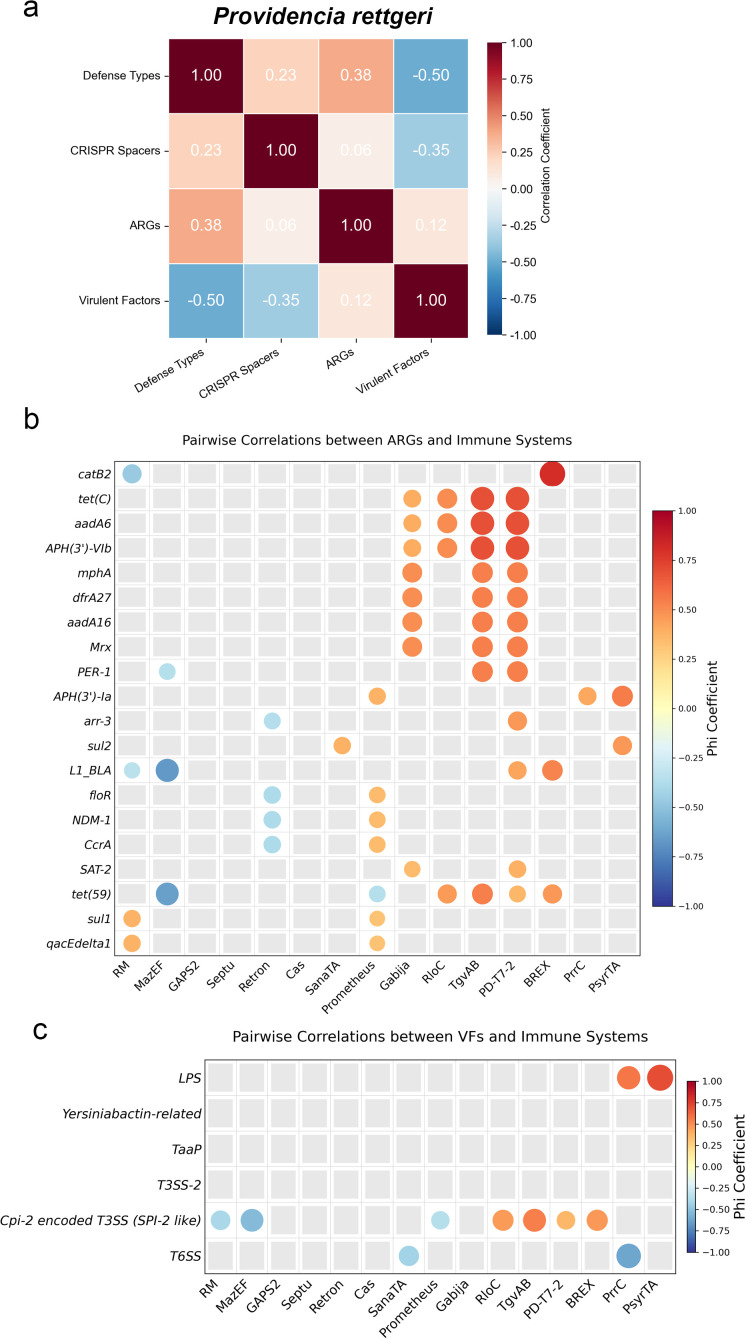
Correlation analysis between defense systems and antimicrobial resistance genes (ARGs) or virulence factors (VFs) in *P. rettgeri*. **(A)** Correlation heatmap showing pairwise relationships among key variables: total number of defense system types, CRISPR spacer count, ARG count, and VF count. **(B, C)** Display pairwise correlation matrices using the Phi coefficient, with circle size and color intensity reflecting the strength and direction of association. Only values with a *p*-value less than 0.1 are displayed in the figure.

To investigate whether defense systems are associated with antimicrobial resistance and virulence, we performed correlation analyses between the total number of defense system types, CRISPR spacer count, ARG count, and VF count in *P. stuartii* and *P. rettgeri*, respectively (**Figures 7A**, **8A**, all data are provided in **Supplementary Data 4**). The results reveal species-specific patterns. In *P. stuartii*, a positive correlation is observed between the number of defense system types and ARGs (r = 0.59), suggesting that strains with more diverse immune repertoires tend to harbor a greater number of ARGs. The association is further described by a negative correlation between defense system diversity and VF count (r = −0.82), indicating a potential trade-off between immune complexity and virulence gene acquisition. Notably, CRISPR spacer count shows a positive correlation with VF count (r = 0.90). In contrast, *P. rettgeri* exhibits a weaker but still positive correlation between defense types and ARGs (r = 0. 38), while showing a negative correlation with VFs (r = −0.50). These findings suggest that although both species show some degree of integration between defense and resistance traits, *P. stuartii* displays a more pronounced pattern of co-enrichment of defense and resistance, coupled with a potential antagonism between immunity and virulence.

In **Figures 7B, C**, we present the correlation analysis between some defense systems identified in *P. stuartii* and specific ARGs or VFs. Similarly, **Figures 8B, C** depict these relationships for *P. rettgeri*. Each point in the figures represents a pairwise comparison of a particular defense system with an individual ARG or VF, using the Phi coefficient to quantify the strength and direction of association. For *P. stuartii*, several defense systems exhibit strong correlations with certain ARGs, suggesting potential co-occurrence patterns within the genome. Conversely, a few defense systems display negative correlations hinting at possible antagonistic relationships or genetic linkage constraints. In *P. rettgeri*, the overall pattern is less pronounced but follows a similar trend, with weaker but still observable correlations between selected defense systems and both ARGs and VFs. These findings provide insight into the genomic architecture of defense, resistance, and virulence traits, highlighting the complex interplay among them.

While the pairwise correlation analyses reveal several statistically significant associations, these findings should be interpreted with caution due to underlying genomic architecture and evolutionary dynamics. Notably, ARGs are frequently observed in clusters, often co-occurring within MGEs such as plasmids, transposons, or integrative and conjugative elements. Consequently, the observed correlations between defense systems and ARGs could reflect genomic co-localization on shared mobile platforms, rather than a direct functional or selective linkage. Similarly, the negative correlation between defense systems and VFs may arise from genomic space constraints, fitness trade-offs, or differential niche adaptation, rather than direct antagonism. Therefore, while the statistical associations provide valuable hypotheses about potential interactions among defense, resistance, and virulence traits, they likely capture indirect signals shaped by genome plasticity and mobile element dynamics. To determine whether these correlations have true biological significance, future studies should integrate genomic context analysis, transcriptomic data, and experimental validation.

### Gain and loss of genes during the evolution of defense systems

The evolution of defense systems in bacteria is a dynamic process marked by both the gain and loss of gene families, which are critical for survival against foreign genetic elements such as bacteriophages and plasmids. Previous studies have highlighted that the acquisition of new defense mechanisms often occurs through HGT, enabling rapid adaptation to environmental pressures. Conversely, the loss of certain defense-related genes may be driven by fitness costs associated with maintaining these systems or due to changes in selective pressures over time. In this study, we calculated the gain and loss events within defense system gene families by employing comparative genomic analyses and ancestral state reconstruction methods. By understanding the dynamics of gene gain and loss, we can better comprehend how bacteria evolve their defensive capabilities in response to changing environments, providing insights into the complex interplay between microbial communities and their biotic and abiotic surroundings.

To investigate the evolutionary dynamics of defense systems in *Providencia*, we reconstructed gene gain and loss events across the phylogeny of *P. stuartii* and *P. rettgeri* using a phylogenomic framework. OrthoFinder was first employed to cluster protein-coding genes into orthogroups, enabling the inference of gene family presence/absence profiles across strains. Subsequently, the COUNT software was applied under a maximum-likelihood birth-death model to estimate per-gene-family rates of gain and loss, accounting for lineage-specific evolutionary processes. This approach allows for the modeling of gene content evolution by treating gains and losses along the branches of the species tree (All data are provided in **Supplementary Data 5**).

Our results reveal distinct evolutionary trajectories between defense systems and the whole genome in both species. In *P. stuartii*, defense systems exhibit experience a higher rate of gene gain (mean: 1.90 × 10^-2^) compared to the whole genome (1.10 × 10^-2^), yet they also undergo substantial gene loss (3.78 × 10^-2^), which—although slightly lower than the whole-genome loss rate (4.91 × 10^-2^) (**Table 2**), resulting in a net loss trend. This suggests that while *P. stuartii* actively acquires new defense genes, it simultaneously discards others at a high rate, indicative of a dynamic, high-turnover immune arsenal. In contrast, *P. rettgeri* displays a higher average gain rate in defense systems, driven by an elevated gain rate (5.01 × 10^-2^), nearly double its whole-genome gain (2.62 × 10^-2^), and a low loss rate (5.96 × 10^-2^), which is only about one-third of its genomic loss rate (1.74 × 10^-2^) (**Table 3**). This indicates that, unlike *P. stuartii*, *P. rettgeri* is undergoing expansion and stabilization of its defense repertoire, possibly reflecting stronger selective pressures to maintain diverse immunity mechanisms. Collectively, these findings demonstrate that defense systems in *Providencia* are subject to high evolutionary turnover, but with species-specific strategies: *P. stuartii* maintains a dynamic, high-turnover, replaceable defense repertoire, whereas *P. rettgeri* favors net acquisition and retention of defense genes. This divergence highlights the distinct evolutionary paths taken by these closely related pathogens in adapting their immune arsenals.

**Table 2:** Genome dynamics in defense systems in all studied *Providencia stuartii*.

Strain	Defense system		Whole genome	
	Gain	Loss	Gain	Loss
GCA000259175	2.92E-02	4.67E-02	2.00E-02	5.97E-02
GCA000754345	2.49E-04	4.81E-04	3.70E-03	4.66E-03
GCA000783455	7.12E-02	3.47E-02	2.44E-02	5.03E-02
GCA001558855	4.01E-02	9.57E-02	1.93E-02	9.05E-02
GCA001888205	8.12E-03	1.06E-02	1.75E-02	2.10E-02
GCA002947315	7.27E-02	7.60E-02	1.81E-02	6.16E-02
GCA002983665	5.22E-04	2.32E-02	7.18E-03	4.92E-02
GCA008693805	1.63E-02	2.79E-02	1.26E-02	3.10E-02
GCA010320365	1.15E-03	2.56E-02	8.67E-03	2.97E-02
GCA010669105	8.73E-02	1.43E-02	1.16E-02	3.20E-02
GCA016128115	2.49E-04	4.81E-04	1.99E-03	1.18E-02
GCA018128385	1.86E-02	6.49E-03	4.04E-03	2.95E-02
GCA023066315	2.94E-02	3.37E-01	8.65E-03	6.25E-01
GCA023520575	1.63E-02	5.72E-02	3.67E-02	5.16E-02
GCA023547145	1.81E-02	1.21E-01	2.27E-02	5.48E-02
GCA023970715	9.69E-02	1.30E-01	6.11E-02	1.32E-01
GCA027286025	3.37E-02	6.71E-02	1.36E-02	6.52E-02
GCA027286045	4.43E-09	2.80E-07	3.39E-08	4.01E-07
GCA027286065	9.67E-09	1.01E-02	2.79E-03	2.43E-02
GCA029277825	2.99E-09	4.66E-08	1.44E-04	1.87E-04
GCA029277985	9.47E-07	1.45E-06	3.40E-04	4.58E-03
GCA029278185	2.99E-09	4.66E-08	1.49E-04	1.92E-04
GCA030179155	1.64E-03	1.87E-02	1.53E-02	2.41E-02
GCA035747985	2.75E-02	1.64E-02	8.68E-03	3.91E-02
GCA038069215	9.30E-09	1.00E-06	8.50E-05	8.90E-04
GCA042165595	9.30E-09	1.00E-06	6.06E-03	1.43E-03
GCA046116575	1.49E-03	3.92E-02	1.07E-02	1.83E-02
GCA050158205	5.65E-05	5.31E-05	5.90E-04	4.62E-03
GCA051549715	5.65E-05	5.31E-05	2.35E-04	1.14E-03
GCA963855485	3.12E-07	1.38E-02	8.34E-04	1.96E-03
GCA963855525	1.66E-02	1.14E-05	2.49E-03	2.30E-03
Average	1.90E-02	3.78E-02	1.10E-02	4.91E-02

**Table 3:** Genome dynamics in defense systems in all studied *Providencia rettgeri*.

	Defense system		Whole genome	
Strain	Gain	Loss	Gain	Loss
GCA000314835	4.36E-02	2.50E-04	2.98E-02	2.89E-02
GCA001874625	6.91E-02	7.63E-03	6.08E-02	6.90E-03
GCA002984195	1.23E-01	3.28E-04	2.56E-02	1.04E-02
GCA003204135	3.60E-02	9.17E-03	4.20E-02	1.35E-02
GCA010318885	1.93E-01	4.16E-03	4.75E-02	2.07E-02
GCA010319105	1.38E-08	2.39E-07	9.77E-08	1.59E-04
GCA010319405	1.38E-08	2.39E-07	4.18E-04	1.44E-04
GCA010320145	2.02E-02	9.78E-03	4.93E-02	1.47E-02
GCA013255915	4.85E-08	6.23E-10	2.09E-08	4.10E-07
GCA013283975	4.70E-02	4.24E-02	1.79E-02	3.09E-01
GCA013423885	7.70E-02	1.81E-02	2.63E-02	7.27E-03
GCA013694385	3.38E-04	8.02E-05	1.55E-02	1.70E-03
GCA013702025	5.18E-05	1.26E-06	1.02E-03	1.06E-03
GCA013702245	6.00E-02	9.46E-03	1.46E-02	1.14E-02
GCA013702265	1.54E-01	2.53E-02	5.30E-02	7.09E-02
GCA014394705	4.85E-08	6.23E-10	1.58E-03	5.33E-09
GCA014489375	8.96E-02	2.26E-02	3.23E-02	1.05E-02
GCA015571575	7.97E-02	4.66E-02	4.79E-02	1.13E-02
GCA016406205	4.55E-06	5.39E-08	4.89E-04	2.61E-07
GCA018771265	4.55E-06	5.39E-08	1.43E-06	1.24E-03
GCA018861215	1.12E-08	8.68E-03	1.78E-03	1.45E-02
GCA018861235	1.19E-05	1.57E-08	1.79E-04	3.31E-04
GCA018861255	1.20E-05	6.97E-09	3.44E-04	1.28E-03
GCA019047885	7.28E-02	2.75E-02	2.30E-02	1.47E-02
GCA019048105	5.27E-02	4.65E-04	4.53E-02	1.89E-02
GCA019048545	7.00E-02	2.45E-03	1.55E-02	7.25E-03
GCA019890815	3.61E-02	5.61E-04	4.73E-02	7.75E-03
GCA020683065	6.72E-03	1.01E-03	1.33E-02	7.63E-03
GCA020808945	3.53E-02	1.42E-04	3.69E-02	4.26E-03
GCA020985345	6.80E-02	2.75E-03	1.34E-02	4.51E-03
GCA022846595	2.54E-02	4.58E-05	3.68E-02	7.89E-04
GCA023184555	3.54E-02	2.31E-03	1.51E-02	1.63E-02
GCA023205015	1.37E-01	1.26E-03	7.60E-02	3.16E-02
GCA023650895	3.38E-04	8.02E-05	5.92E-03	2.47E-03
GCA025583505	1.22E-01	2.63E-04	4.61E-02	1.25E-02
GCA025916175	1.19E-01	1.60E-03	4.47E-02	1.67E-02
GCA029011985	5.18E-05	1.26E-06	1.83E-03	6.05E-03
GCA034330845	5.79E-04	8.88E-05	1.78E-02	1.81E-03
GCA038442735	1.21E-01	5.93E-05	9.50E-02	4.70E-03
GCA040208285	1.04E-01	2.18E-03	4.01E-02	7.54E-03
GCA041075285	1.05E-01	2.87E-03	4.25E-02	2.00E-02
GCA042142345	5.79E-04	8.88E-05	1.50E-02	7.89E-03
Average	5.01E-02	5.96E-03	2.62E-02	1.74E-02

### Functional validation confirms anti-phage activity of *Providencia*-encoded Gabija and Septu systems

To experimentally test whether these computationally identified systems are functional, we selected two well characterized systems, Gabija from *P. stuartii* (GCA_010669105) and Septu from *P. rettgeri* (GCA_010318885), for heterologous expression and phage challenge assays in *E. coli DH5 alpha* (**Supplementary Data 6**).

**Figure 9:**
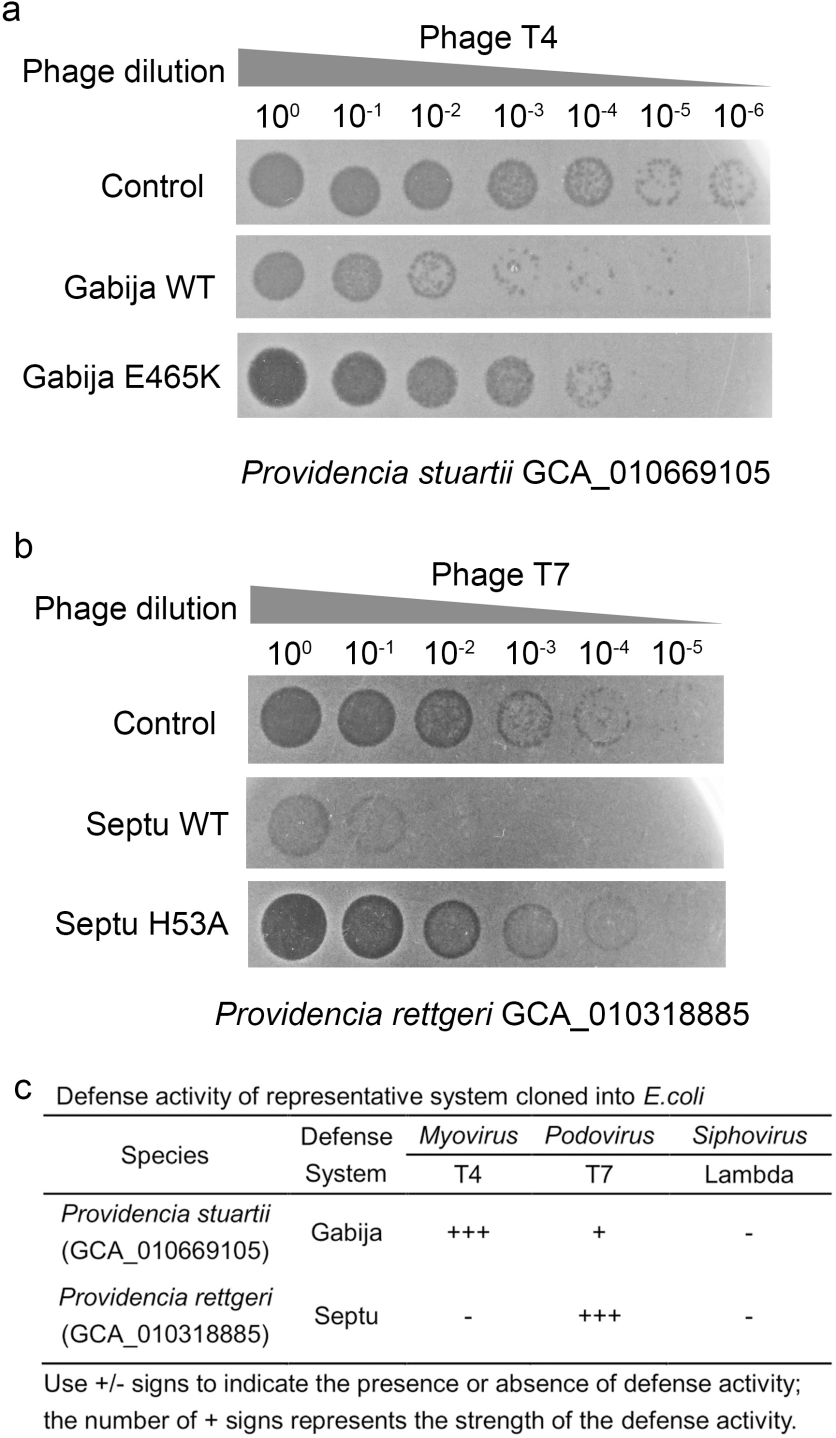
Functional validation of Gabija and Septu defense systems from *Providencia* against bacteriophages. **(A)** Phage T4 plaque assay showing the anti-phage activity of the Gabija system in *P. stuartii* (GCA_010669105). The wild-type (WT) Gabija system effectively restricts T4 phage propagation, as evidenced by reduced plaque formation at high dilutions. In contrast, the GajA E465K mutant exhibits significantly diminished defense activity, allowing increased plaque development. Control represents unmodified strain without defense system. **(B)** Phage T7 plaque assay demonstrating the function of the Septu system in *P. rettgeri* (GCA_010318885). The WT Septu system confers strong resistance to T7 phage, with minimal plaque formation across all dilutions. The PtuB H53A mutant shows loss of defense activity, resulting in robust plaque growth comparable to the control. Control indicates the absence of the Septu system. **(C)** Summary of defense activity for representative Gabija and Septu systems cloned into *E*. *coli*. Activity against *Myoviridae* (T4), *Podoviridae* (T7), and *Siphoviridae* (λ) phages is indicated by +/− signs: “+” denotes presence and number of “+” reflects relative strength of defense (+++, strong; +, moderate; −, no activity).

Gabija and Septu were chosen for validation due to their well-characterized molecular mechanisms. Both systems have been extensively studied in *E. coli*, where they confer robust immunity against diverse phages through distinct abortive infection or DNA-targeting strategies. As shown in **Figure 9A**, the wild-type Gabija system from *P. stuartii* strongly inhibited plaque formation by T4 phage, confirming its anti-phage activity. Introducing a single point mutation (E465K) in the GajA subunit, a residue conserved in the predicted ATPase domain, significantly attenuated this defense. Similarly, the Septu system from *P. rettgeri* conferred potent resistance against T7 phage (**Figure 9B**), while the PtuB H53K mutant lost nearly all protective capacity. Broader phage profiling revealed that Gabija exhibited strong activity against T4 (+++), weak inhibition of T7 (+), and no effect on λ phage (−), whereas Septu was highly effective against T7 (+++) but inactive against both T4 and λ (**Figure 9C**). These results demonstrate that *Providencia*-derived defense systems retain functionality in a heterologous host and display phage -specific activity patterns consistent with their known biological roles.

Notably, although *Providencia* belongs to the *Enterobacteriaceae* family and shares close phylogenetic proximity with *E. coli*, the Gabija system from *P. stuartii* GCA_010669105 exhibits structural divergence. While core domains (e.g., TOPRIM in GajA) are conserved, the GajA protein is extended to 736 amino acids, with a longer C-terminal region than the well characterized homolog; however, we do not know whether the C-terminal has any function or not. This uncharacterized segment may reflect *Providencia*-specific adaptations. However, comprehensive functional dissection of such features requires phages naturally infecting *Providencia*, which are currently unavailable for us.

## Discussion

This study presents a comparative genomic analysis of defense systems in *P. stuartii* and *P. rettgeri*, revealing species-specific patterns in their occurrence, diversity, and evolutionary dynamics. Both species possess a wide array of defense mechanisms, yet differ markedly in repertoire composition and genomic stability. *P. stuartii* exhibits a higher diversity of defense systems, greater evolutionary turnover, and stronger associations with ARGs. CRISPR-Cas systems are variably present and show structural divergence in spacer and repeat, suggesting functional differentiation between the two species. Notably, the relationships between defense systems, antibiotic resistance, and VFs are complex and context-dependent, indicative of intertwined evolutionary trajectories. Together, these findings highlight distinct adaptive strategies underlying bacterial immunity in these emerging opportunistic pathogens.

The defense arsenal of *Providencia* pathogens is characterized by a dominant core of RM and CRISPR-Cas systems accompanied by a diverse array of auxiliary mechanisms, reflecting a multi-layered strategy for combating MGEs (26). Cross-tool validation (DefenseFinder, PADLOC) and expanded assembly-level analysis (contigs, scaffolds) confirm the consistency of this core architecture: the overwhelming prevalence of RM systems in both *P. stuartii* and *P. rettgeri* (**Figures 2A, B**; **Supplementary Figures 2A–D**) aligns with their status as the most widespread prokaryotic defense mechanism, consistently reported across bacterial phyla as a primary barrier against foreign DNA (27). Similarly, the high abundance of CRISPR-Cas, particularly Class 1 Type I-F, mirrors findings in other pathogens, where this subtype is frequently associated with robust anti-phage activity (28). Notably, the near-exclusive presence of Cas Type I-F in *Providencia* (detected by both tools), to the apparent exclusion of other common subtypes like I-E or II-A, suggests a specific evolutionary trajectory favoring a single, highly optimized adaptive immune system (29). The enrichment of non-CRISPR systems such as GAPS2, and RM further underscores the reliance on innate, often abortive infection-based defenses, which have been increasingly recognized for their synergistic potential with other systems (28). The clustering of defense genes into genomic islands (**Figures 4C, D**), particularly in *P. stuartii*, echoes the concept of “defense islands” previously described in diverse bacteria, where co-localized systems may facilitate horizontal transfer and functional cooperation (12, 30). Expanded sampling via contig/scaffold data revealed that core defense system profiles (RM, CRISPR-Cas) are conserved across assembly types, though rare systems show greater variability, supporting the utility of contig/scaffold assemblies when complete genomes are scarce. Altogether, the composition and organization of defense systems in *Providencia* highlight a balance between conserved, core immunity and flexible, modular innovation, likely underpinning its resilience in clinical environments.

Despite the presence of CRISPR-Cas systems in a subset of strains, both *P. stuartii* and *P. rettgeri* exhibit remarkably limited diversity in effector architecture, harboring almost exclusively the Class 1, Type I-F system, a subtype commonly associated with plasmid-targeting activity and frequently found in Gram-negative *Enterobacteriaceae* family such as *E. coli* and *Pectobacterium atrosepticum* (31, 32). This conserved subtype, paired with our BacMGEnet data, clarifies its functional role: Type I-F’s dominance aligns with its plasmid/phage-targeting capacity, directly shaping *Providencia*’s MGE interaction networks. Notably, while both species typically retain a single Cas operon, they differ significantly in the genomic organization of associated CRISPR arrays. In *P. stuartii*, multiple spatially separated arrays are often observed across the genome, potentially enabling broad surveillance through a single Cas machinery, a configuration reminiscent of “isolated CRISPR arrays” seen in *E. coli* and *Salmonella*, where distant repeats are processed by a common Cas complex (33, 34). This dispersed architecture dovetails with *P. stuartii*’s expanded spacer count: it likely enhances capture of diverse spacers, critical for navigating high-MGE clinical niches. In contrast, *P. rettgeri* predominantly features one or two arrays immediately adjacent to the *cas* genes, suggesting a more common pattern frequently evolved (33). This structural divergence may reflect differing evolutionary strategies: the dispersed architecture in *P. stuartii* could enhance adaptive potential by capturing diverse spacers from mobile elements, whereas the compact organization in *P. rettgeri* may favor regulatory simplicity and functional reliability. Such variation underscores that even within a conserved CRISPR type, genomic context and array topology play critical roles in shaping immune capability and evolutionary trajectory (33, 35). Notably, the frequent loss or fragmentation of *cas* genes in both species parallels patterns seen in multidrug-resistant pathogens, where CRISPR absence correlates with increased acquisition of resistance, suggesting a selective disadvantage in high-antibiotic settings (36).

The spacer-MGE and host-MGE networks (**Figure 6**; **Supplementary Figure 5**) illuminate how *Providencia*’s defense systems shape its ecological interaction, extending beyond individual strain immunity to community-level dynamics. The dense, hub-driven architecture of *P. stuartii*’s network (shared spacers targeting common phages) aligns with its expanded defense system repertoire: frequent phage exposure (driving CRISPR spacer acquisition) likely selects for conserved anti-phage immunity across strains, a pattern consistent with hospital environments where phage predation is pervasive. In contrast, *P. rettgeri*’s sparser network mirrors its reduced CRISPR diversity, suggesting either lower phage pressure or reliance on alternative defenses to mitigate MGE threats. More broadly, integrating defense system composition (RM, CRISPR subtypes) with interaction networks bridges molecular immunity to microbial community ecology, offering a model for studying how pathogens adapt their defenses to fluctuating MGE landscapes.

ARGs and VFs are key determinants of pathogen success and now could be served as prime targets of phage therapy, it is therefore imperative to clarify how these resistance and virulence determinants interplay with bacterial defense systems (37–39). The observed associations between defense system content and ARGs or VFs, align with growing evidence that bacterial immunity and resistance evolution are not independent processes (40). In the groundwater defensome, the same mobile islands that deliver high-turnover accessory immune genes rarely carry antibiotic-resistance determinants, providing a natural example of an inverse link between defense-system load and ARG abundance (41). Similarly, Analysis of 13,000+ chromosomally integrated MGEs shows that defense-rich MGEs simultaneously carry fewer ARGs and fewer virulence genes, demonstrating a single inverse relationship that links high bacterial defense-system load to both reduced antibiotic-resistance potential and diminished virulence capacity (42). Our observation of a positive correlation between defense systems and ARGs, contrary to these pan-environmental studies. This apparent discrepancy may stem from the critical influence of ecological and genomic context on bacterial evolutionary dynamics. Their results likely reflect environments with lower antibiotic pressure, where defense systems function primarily as barriers to uncontrolled genetic influx. In contrast, clinical and hospital-associated strains, frequently exposed to antibiotics, may experience co-selection of defense and resistance genes.

Gene gain and loss are fundamental drivers of prokaryotic genome evolution, shaping the adaptive potential of bacterial lineages by enabling rapid responses to environmental challenges (43). Comparative genomics reveals that defense systems are among the most dynamically evolving modules in prokaryotic genomes, subject to rapid gain and loss driven by the constant turnover of genetic parasites (44). This fluid architecture generates lineage-specific defense islands and continually reshapes the immunity landscape of bacteria and archaea (44). Defense systems experience rapid gain and loss via HGT, often co-mobilized with MGEs, which can transiently increase HGT rates rather than suppress them (45). Defense genes are among the most rapidly exchanged commodities in microbial genomes, exhibiting an average gain-and-loss flux 1.4-fold higher than the genomic background and recurring in the same locus through repeated, MGE-mediated birth-and-death events (46). In *Ralstonia solanacearum*, defense systems against phages and plasmids exhibit a dynamic evolutionary pattern characterized by a net gain of defense genes, contrasting with the overall trend of gene loss in the rest of the genome (47). Together, these findings establish that defense systems do not evolve under stable inheritance but instead follow a highly dynamic trajectory shaped by MGEs and host-parasite coevolution. In line with this broader evolutionary paradigm, our analysis of *Providencia* genomes reveals extensive gene turnover in defense systems, consistent with their classification as accessory genome components under strong diversifying selection. Notably, while both *P. stuartii* and *P. rettgeri* exhibit dynamics of gain and loss (**Tables 2**, **3**), the directionality of change differs between species: *P. stuartii* displays a high-turnover model, reflecting frequent replacement and remodeling of defense repertoires. In contrast, *P. rettgeri* shows a bias toward net gene gain, mirroring the expansionist pattern observed in *Ralstonia solanacearum* (47) and suggesting intensified selection for novel defense acquisition. This divergence underscores that closely related pathogens can adopt distinct evolutionary strategies, ranging from defense system recycling to sustained expansion. Thus, while the dynamic nature of defense systems is conserved across bacteria, the net outcome of their evolution is fine-tuned to lineage-specific ecological pressures.

While this study provides a comprehensive pan-genomic view of defense system in *Providencia*, several limitations still exist. First, the reliance on genomes and automated annotation may lead to incomplete or erroneous identification of defense loci, particularly for fragmented or divergent systems that require manual curation and functional validation. Second, the observed correlations between defense systems, ARGs, and VFs are based on genomic co-occurrence and do not establish causality. Experimental data, such as those provided by RNA-seq analyses, could be used to determine whether these systems are actively expressed under relevant environmental or host conditions (48). Third, the evolutionary inferences drawn from phylogenetic comparative methods assume neutral background evolution, yet selection on linked sites or population structure could bias gain-loss reconstructions. Moreover, expanding comparative analyses to include closely related genera and diverse ecological niches could reveal broader principles governing the trade-offs between immunity, resistance, and virulence in opportunistic pathogens.

Our functional validation of *Providencia*-encoded Gabija and Septu systems not only confirms their anti-phage activity in a heterologous host but also situates our findings within the rapidly expanding landscape of prokaryotic innate immunity. The discovery of these two systems traces back to the landmark pangenomic screen by Doron et al. (2018), which first identified Gabija and Septu as widespread, previously uncharacterized defense modules clustered in genomic “defense islands” (49). Since then, structural and mechanistic studies have revealed that both systems operate through tightly regulated, multi-subunit complexes: Gabija functions as a nucleotide-sensing DNA endonuclease whose activity is unleashed upon phage-induced ATP/dNTP depletion (50–52), while Septu forms an inflammasome-like PtuA6:PtuB2 oligomer that degrades single-stranded DNA upon activation (53, 54). Our observation that point mutations in conserved residues, GajA E465K and PtuB H53K, abolish defense aligns with these models, as E465 resides within the ATPase/Toprim regulatory interface critical for Gabija activation (52, 55) and H53 likely contributes to PtuB’s nuclease function or complex assembly (53).

Notably, despite high conservation of core domains, the *P. stuartii* Gabija system harbors a longer C-terminal region than its homologs reported. Given that GajB has been shown to modulate substrate specificity and enhance plasmid cleavage in other species (56), this extended region may fine-tune the immune response in *Providencia*. Intriguingly, recent work suggests Gabija discriminates self from non-self not by sequence motifs but by sensing the absence of host RecBCD-mediated DNA repair on linear phage genomes (57); whether such a mechanism operates in *Providencia*, which possesses divergent DNA repair machinery, remains unknown.

Critically, our assays relied on model phages (T4, T7, λ) in *E. coli*, which, while enabling initial validation, may overlook the full defensive repertoire of *Providencia*. Native phages co-evolving with *Providencia* could engage distinct evasion strategies (e.g., encoding Gad1-like anti-defense proteins that encapsulate Gabija (50)) or trigger alternative activation pathways. Moreover, emerging evidence shows that some defense systems, including Retron-Septu, are maintained in an inactive state until disassembly releases active effectors, suggesting that static heterologous expression may underestimate regulatory complexity (54). Future work employing *Providencia*-specific phages, native expression contexts, and structural interrogation of *Providencia*-derived complexes will be essential to uncover whether these systems have evolved unique adaptations reflective of their ecological niche, particularly in clinical settings where phage pressure intersects with antibiotic selection.

This study highlights that defense systems in *Providencia* are not static genomic elements but dynamic components. Our findings reinforce the emerging view that microbial defense is not merely a binary trait but part of an integrated genomic ecosystem, in which interactions with MGEs drive the co-evolution of resistance, virulence, and immunity. As such, understanding the regulatory and evolutionary logic of defense systems may offer new avenues for predicting pathogen evolution and designing phage-based therapies. Future models of bacterial adaptation must therefore incorporate defense architecture as a key variable, bridging genomics, ecology, and function to unravel the full complexity of host-parasite arms races in the microbial world.

## Materials and methods

### Data collection

All complete genome sequences of *P. stuartii* and *P. rettgeri* were retrieved from the NCBI GenBank database (https://www.ncbi.nlm.nih.gov/genbank/) in 2025-12-29. A total of 31 P*. stuartii* and 42 P*. rettgeri* isolates with complete genomes or chromosome available were included in this study. To broaden our analysis beyond complete genomes, we retrieved all publicly available genomic assemblies from NCBI, including contig- and scaffold-level sequences. This yielded 429 contigs and 50 scaffolds for *P. stuartii*, and 334 contigs and 244 scaffolds for *P. rettgeri*. All assemblies were processed with DefenseFinder under default parameters to identify and classify defense systems. Only complete genomes were selected for most analysis to ensure high genomic integrity and to minimize assembly-related artifacts in the identification and genomic context analysis of defense systems. All accession numbers for the analyzed genomes are provided in **Supplementary Table 1**. For downstream analyses requiring different genomic formats (e.g., nucleotide sequences in FASTA, annotated features in GFF or GBK), corresponding files were downloaded directly from NCBI without re-annotation to maintain consistency with the reference records and to ensure reproducibility of the analyses. However, due to differences in file availability across NCBI repositories and formats, slight discrepancies in isolate representation may occur between individual bioinformatics workflows.

### Identification and characterization of defense systems

Defense systems were identified using DefenseFinder (version 2.2) with default parameters, a widely used tool for the detection and classification of known antiviral defense systems in prokaryotic genomes (27, 58). The analysis was performed on the complete genome sequences of 31 P*. stuartii* and 42 P*. rettgeri* isolates. DefenseFinder was run for each isolate, and results were aggregated for comparative analysis. To visualize the distribution and composition of defense systems across strains, Python (v3.9) libraries such as pandas, matplotlib, and seaborn are used for data processing and plotting.

A complementary analysis using PADLOC (v2.0.0) on the same set of complete *P. stuartii* and *P. rettgeri* genomes previously analyzed with DefenseFinder (59, 60). PADLOC was run under default settings to identify defense systems at the subtype level. Results from PADLOC were compared with DefenseFinder outputs to assess concordance in major system detection and to identify potentially missed or underrepresented immune modules.

### Identification and characterization of CRISPR-Cas system

The CRISPR-Cas systems were identified using CRISPRCasFinder (v4.2.21) (61) and CRISPRCasTyper (v1.3) (62) with default parameters, run locally in command-line mode to enable batch processing. CRISPRCasFinder was used to detect both CRISPR arrays and associated *cas* genes across all complete genomes. Only CRISPR arrays with an evidence level of 3 or 4, indicating strong support based on repeat conservation and spacer length, were retained for downstream analysis to ensure high-confidence predictions. Genomic context analysis was performed to determine the physical linkage between CRISPR arrays and *cas* gene clusters. Manual curation was carried out using genome browsers and sequence inspection to confirm the boundaries of arrays and the orientation of flanking genes. The RNA secondary structures of the repeat sequences were predicted using the RNAfold WebServer (available at http://rna.tbi.univie.ac.at/cgi-bin/RNAWebSuite/RNAfold.cgi) with default settings. The minimum free energy (MFE) structure was used to assess the potential for stable stem-loop formation.

### Spacer and MGE network analysis

To investigate the potential interactions between *P. stuartii* and *P. rettgeri* with MGEs (including phages and plasmids), the BacMGEnet computational pipeline (https://github.com/mgtools/BacMGEnet) was employed in this study (63). Briefly, the downloaded genomic data of the two bacterial species was used as input. CRISPR arrays and associated spacers were identified using the integrated method, followed by redundancy removal with CD-HIT-EST to obtain unique spacers. These unique spacers were then queried against the multi-source MGE database (downloaded from BacMGEnet pipeline) integrated by BacMGEnet (encompassing phage databases such as GPD, MVP, RVDB, and mMGE, as well as plasmid databases including COMPASS and PLSDB) using BLASTN. Matches were filtered with criteria of >90% sequence identity, >80% query coverage, and e-value <0.001 to identify potential MGEs containing corresponding protospacers. A greedy algorithm was applied to select non-redundant bacterial hosts and MGEs, which were used to construct Host-MGE and Spacer-MGE interaction networks. Network visualization along with manual verification were conducted using Cytoscape.

### Correlation analysis of ARGs and VFs

To investigate the potential associations between defense systems and genomic features related to pathogenicity, VFs and ARGs were annotated for each *Providencia* genome using data retrieved from the gcPathogen database (https://nmdc.cn/gcpathogen/), a curated resource for pathogen-associated genes in bacterial genomes (64). The presence and abundance of VFs and VRGs were matched to the corresponding isolates based on genome accession ids.

A pairwise correlation analysis was performed between the occurrence profiles of defense system and the presence of individual VFs and ARGs. The analysis was conducted using Phi coefficient (φ), a measure of association for binary variables. To evaluate the association between the overall abundance of defense systems and ARGs/VFs, Spearman’s rank correlation coefficient (ρ) was calculated to assess non-linear relationships, and the corresponding *p*-values were adjusted for multiple testing using the Benjamini-Hochberg procedure to control the false discovery rate (FDR). Correlations with an adjusted *p*-value < 0.1 were considered suggestive of potential biological associations.

### Gene gain and loss

To infer the evolutionary dynamics of defense system gene families in *Providencia*, we applied a probabilistic birth-and-death model implemented in COUNT v10.04 (https://www.iro.umontreal.ca/~csuros/gene_content/count.html) (65). The analysis was based on defense system-specific gene family clustering and a species tree inferred using OrthoFinder (v2.5.4) with default parameters (66). OrthoFinder was used to identify orthogroups from the proteomes of *Providencia* isolates (31 P*. stuartii* and 42 P*. rettgeri* strains available from NCBI GenBank), and the rooted species tree generated by OrthoFinder served as the phylogenetic framework for downstream ancestral state reconstruction. The gene family gain, loss, and duplication rates were estimated using the gain-loss-duplication model under a Poisson distribution, with rate variation across branches modeled using three discrete categories of the gamma distribution to account for heterogeneity in evolutionary rates. Similar results were obtained when using four gamma categories, confirming the robustness of the model. For optimization, 100 independent runs were performed to ensure convergence, with the likelihood threshold set at 0.1. Custom scripts were written in-house to quantify gains and losses of defense system families using COUNT-derived results; these scripts are available in the **Supplementary Materials** (**Supplementary Data 7**). This combined OrthoFinder-COUNT approach enabled the reconstruction of ancestral gene content and the estimation of lineage-specific rates of defense system gene family evolution across the *Providencia* phylogeny.

### Functional validation of Gabija and Septu defense systems

The Gabija and Septu anti-phage defense systems were identified in representative *Providencia* strains: Gabija from *P. stuartii* (GCA_010669105) and Septu from *P. rettgeri* (GCA_010318885). Full genomic coordinates, gene accession IDs, protein sequences, and complete DNA sequences of both systems are provided in **Supplementary Data 6**. For functional assays, the entire defense loci, including 500 bp upstream and 200 bp downstream of the coding regions, were chemically synthesized by Sangon Biotech Co., Ltd. (Shanghai, China) and cloned into the low-copy-number plasmid vector pBR322 under native regulatory elements.

Site-directed mutagenesis was performed to generate loss-of-function variants: GajA E465K in the Gabija system and PtuB H53K in the Septu system. Mutations were introduced using the QuickMutation Site-Directed Mutagenesis Kit (Cat. No. D0206S, Beyotime Biotechnology, China), following the manufacturer’s protocol. All constructs were verified by Sanger sequencing.

Wild-type and mutant defense constructs were transformed into Escherichia coli DH5α cells for phenotypic characterization. Phage plaque assays were conducted as follows: overnight cultures of DH5α harboring each construct were diluted 1:100 in LB medium and grown to mid-log phase (OD600 ≈ 0.5). Cells were mixed with serial dilutions (10^−1^ to 10^−7^) of laboratory-maintained bacteriophages T4 (*Myoviridae*), T7 (*Podoviridae*), or λ (*Siphoviridae*), incubated at 37 °C for 15 min to allow phage adsorption, and then drop plated in agar overlays on LB agar plates. Plaques were visualized after overnight incubation at 37 °C. Defense activity was presented based on plaque formation relative to empty-vector controls: “−” indicates no restriction (plaque morphology identical to control), “+” denotes moderate reduction in plaque number or size, and “+++” represents strong inhibition (few or no plaques even at low phage dilutions).

### Statistical comparisons of defense system

Statistical comparisons of defense system type/subtype counts across bacterial genera and species were conducted using one-way analysis of variance (ANOVA) to assess overall differences between groups. Where significant main effects were detected, Tukey’s honest significant difference (HSD) *post hoc* test was applied to perform pairwise comparisons between *Providencia* (genus level), *P. stuartii, P. rettgeri* (species level), and other target taxa. Significance thresholds were set at *p* < 0.05 and *p* < 0.001. All statistical analyses were implemented in Python using the scipy and statsmodels packages. Full pairwise comparison results are provided in **Supplementary Data 1**.

## Data Availability

The original contributions presented in the study are included in the article/**Supplementary Material**. Further inquiries can be directed to the corresponding author.
