# Supplementary figures and images for: Characterization and diversity of defense systems in Providencia pathogen
Source: Front Immunol. 2026 Feb 12;17:1755933. doi: 10.3389/fimmu.2026.1755933 (PMC12935923; doi:10.3389/fimmu.2026.1755933)

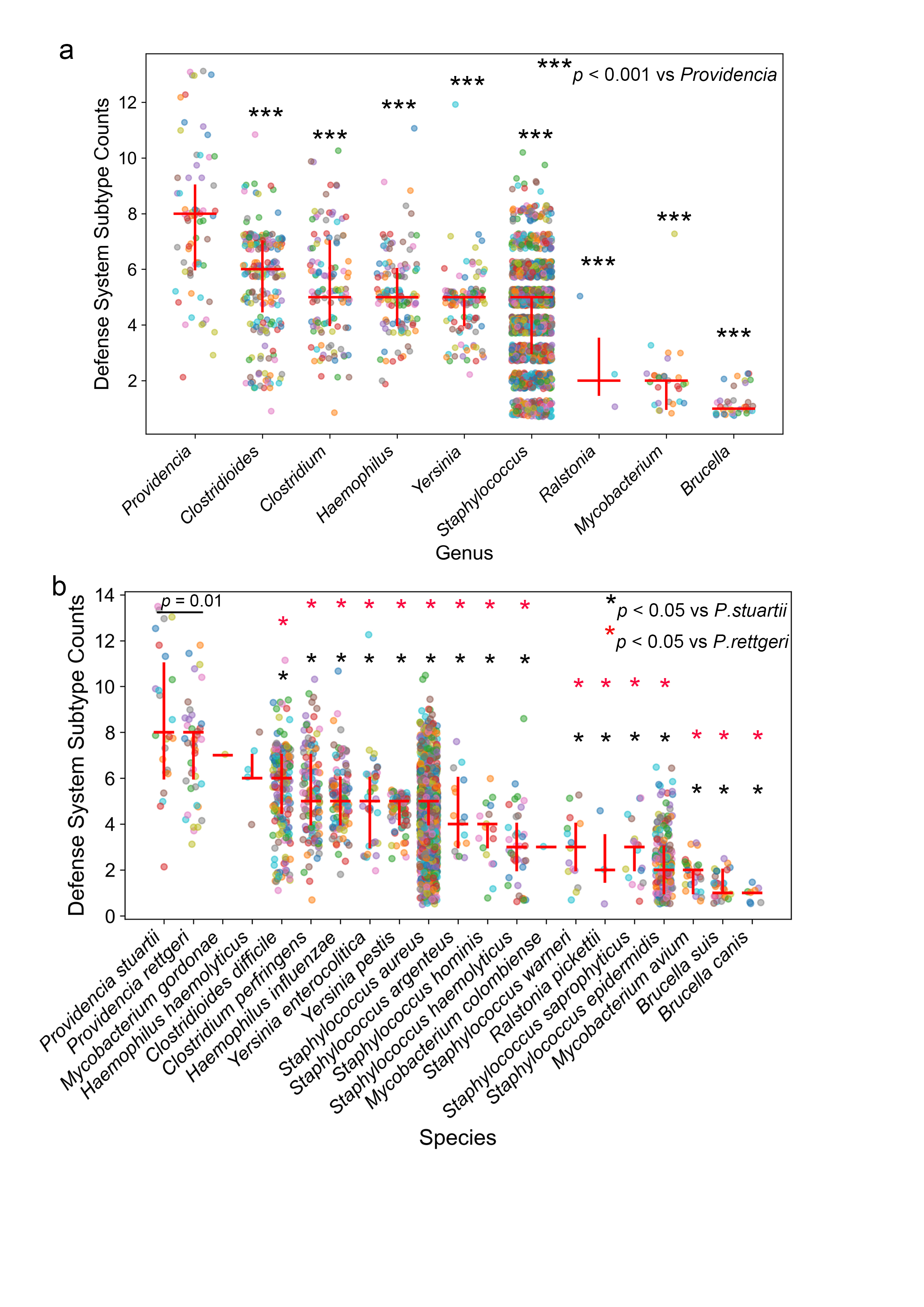

Supplement: Supplementary file 1 [file DataSheet1.zip › Supplementary_Figures/SuppFig1.jpg]

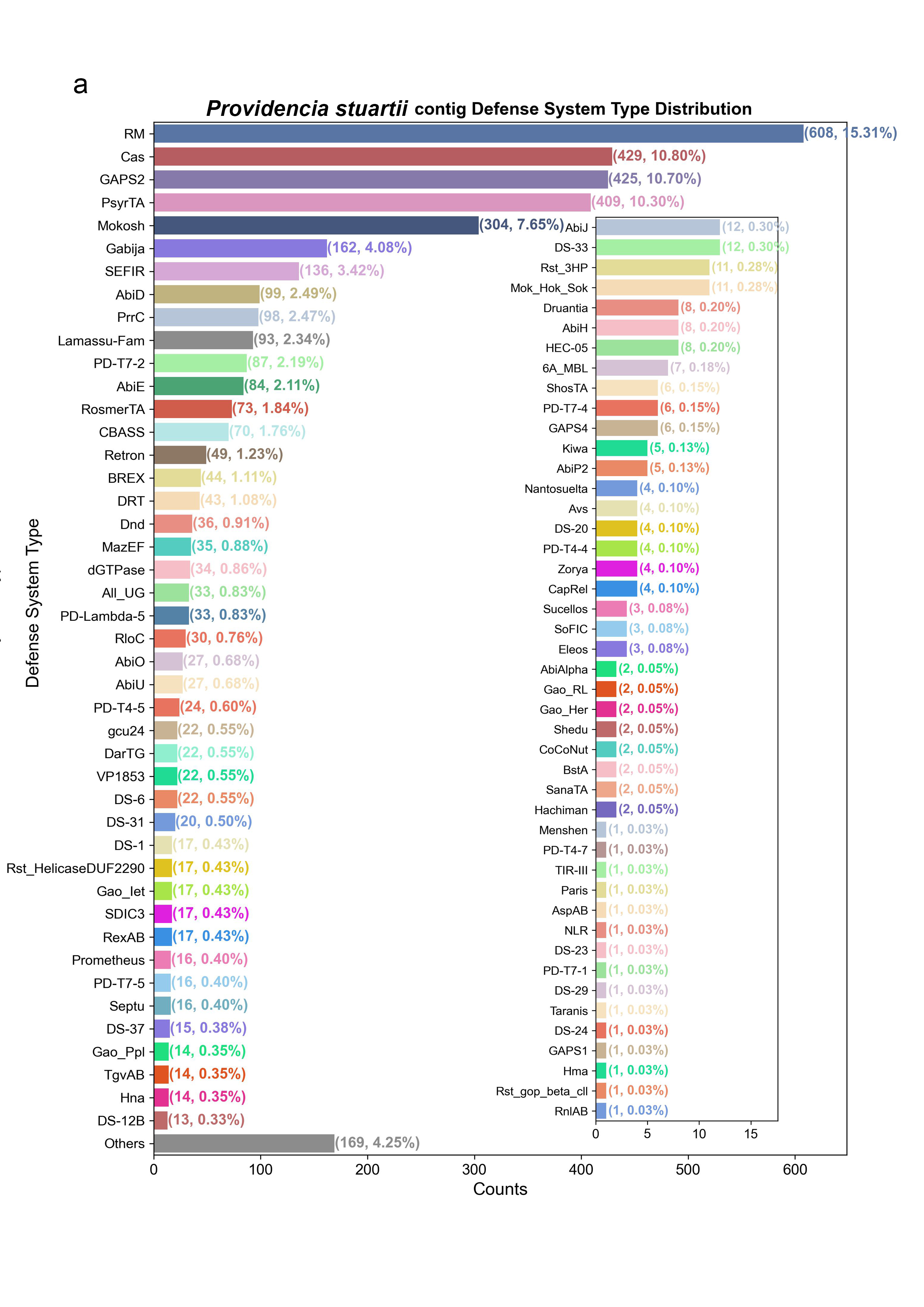

Supplement: Supplementary file 1 [file DataSheet1.zip › Supplementary_Figures/SuppFig2a.jpg]

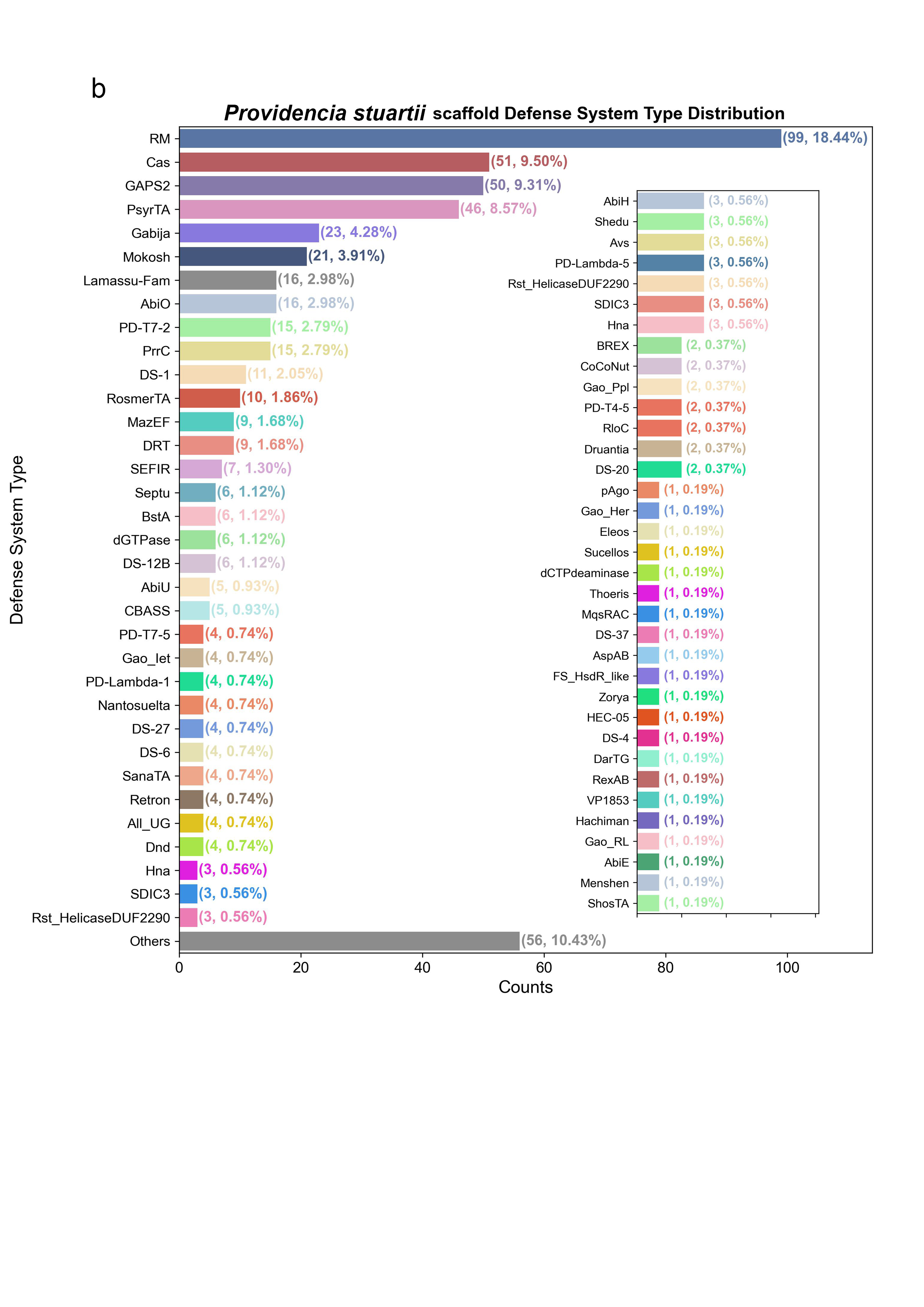

Supplement: Supplementary file 1 [file DataSheet1.zip › Supplementary_Figures/SuppFig2b.jpg]

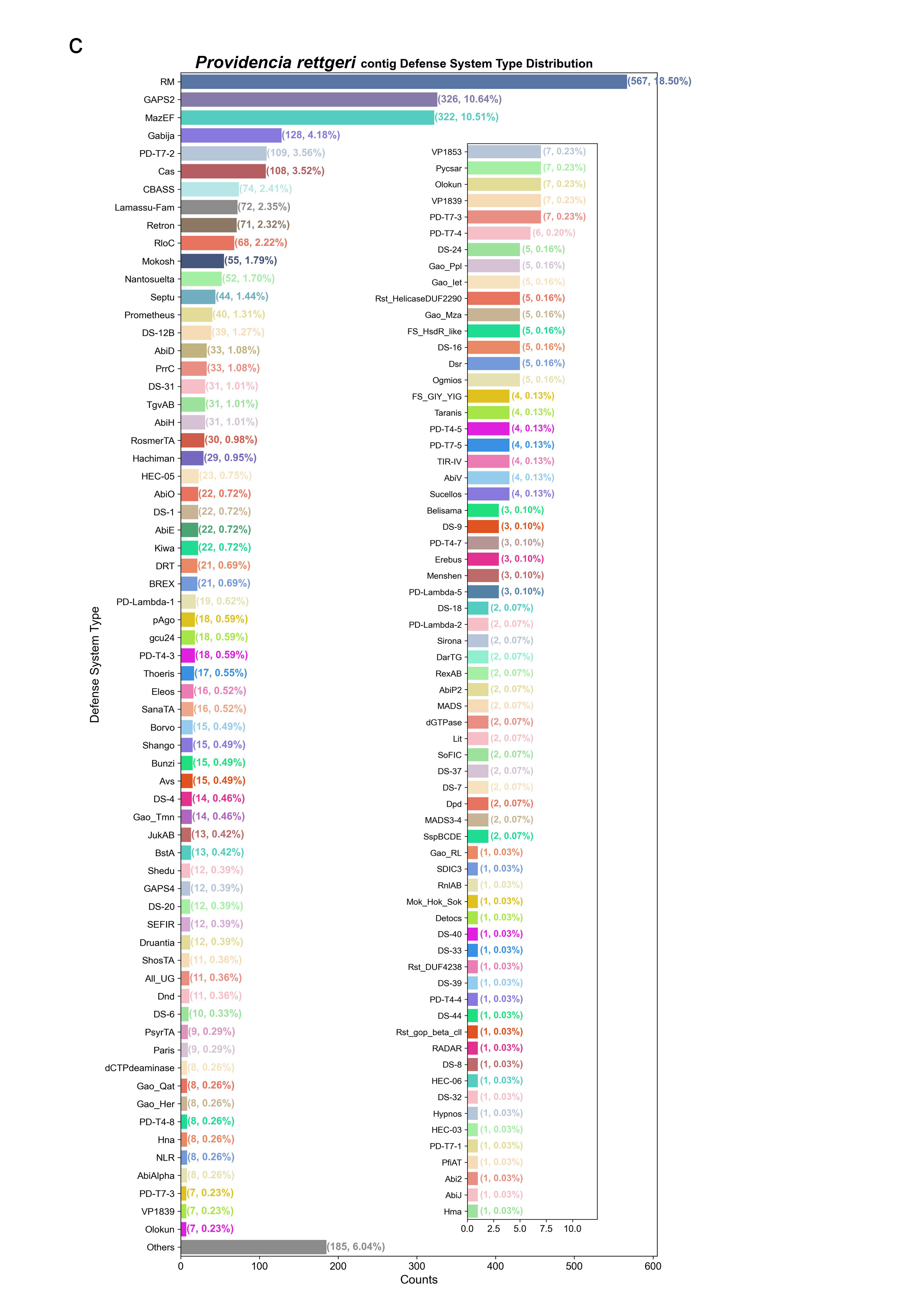

Supplement: Supplementary file 1 [file DataSheet1.zip › Supplementary_Figures/SuppFig2c.jpg]

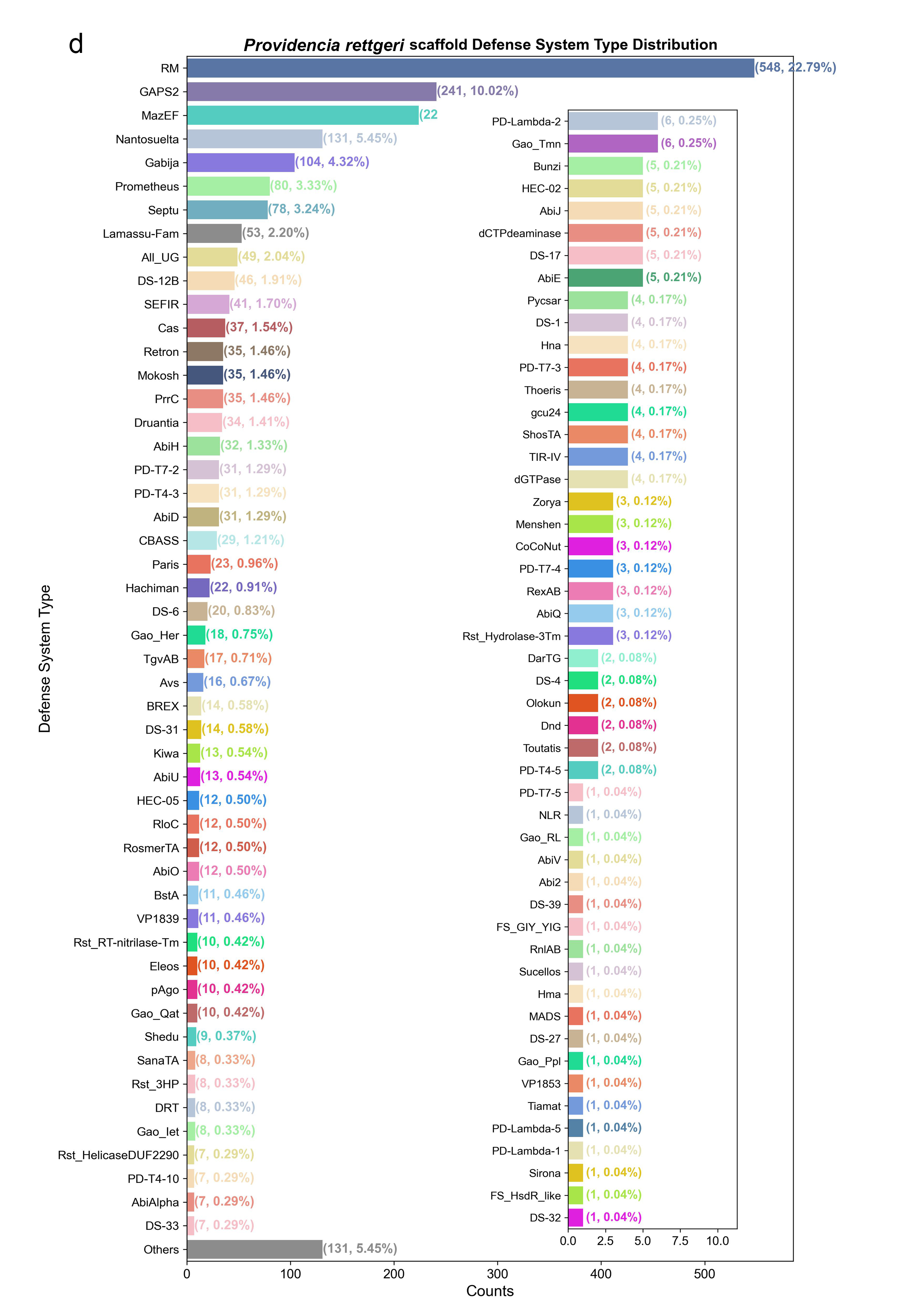

Supplement: Supplementary file 1 [file DataSheet1.zip › Supplementary_Figures/SuppFig2d.jpg]

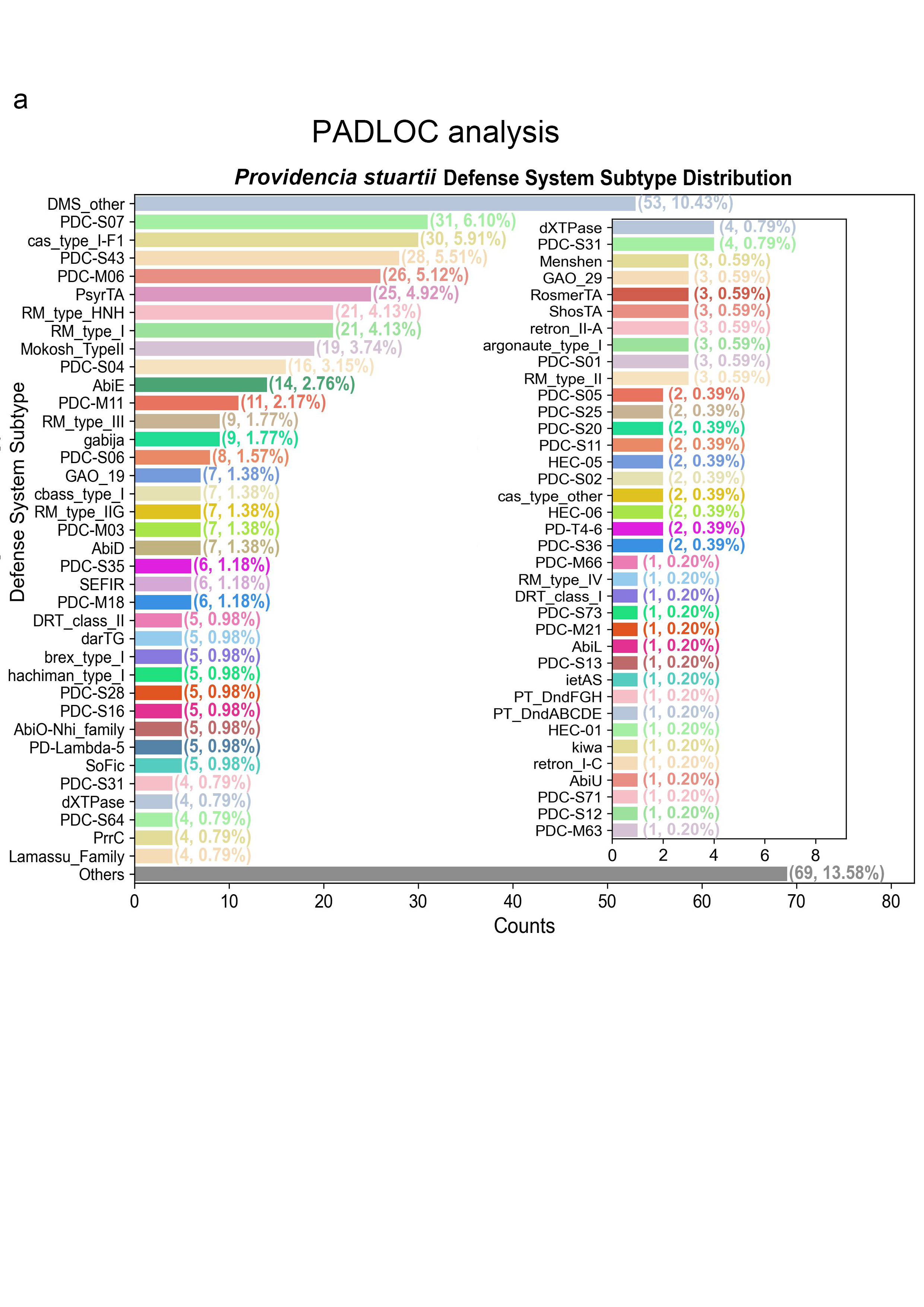

Supplement: Supplementary file 1 [file DataSheet1.zip › Supplementary_Figures/SuppFig3a.jpg]

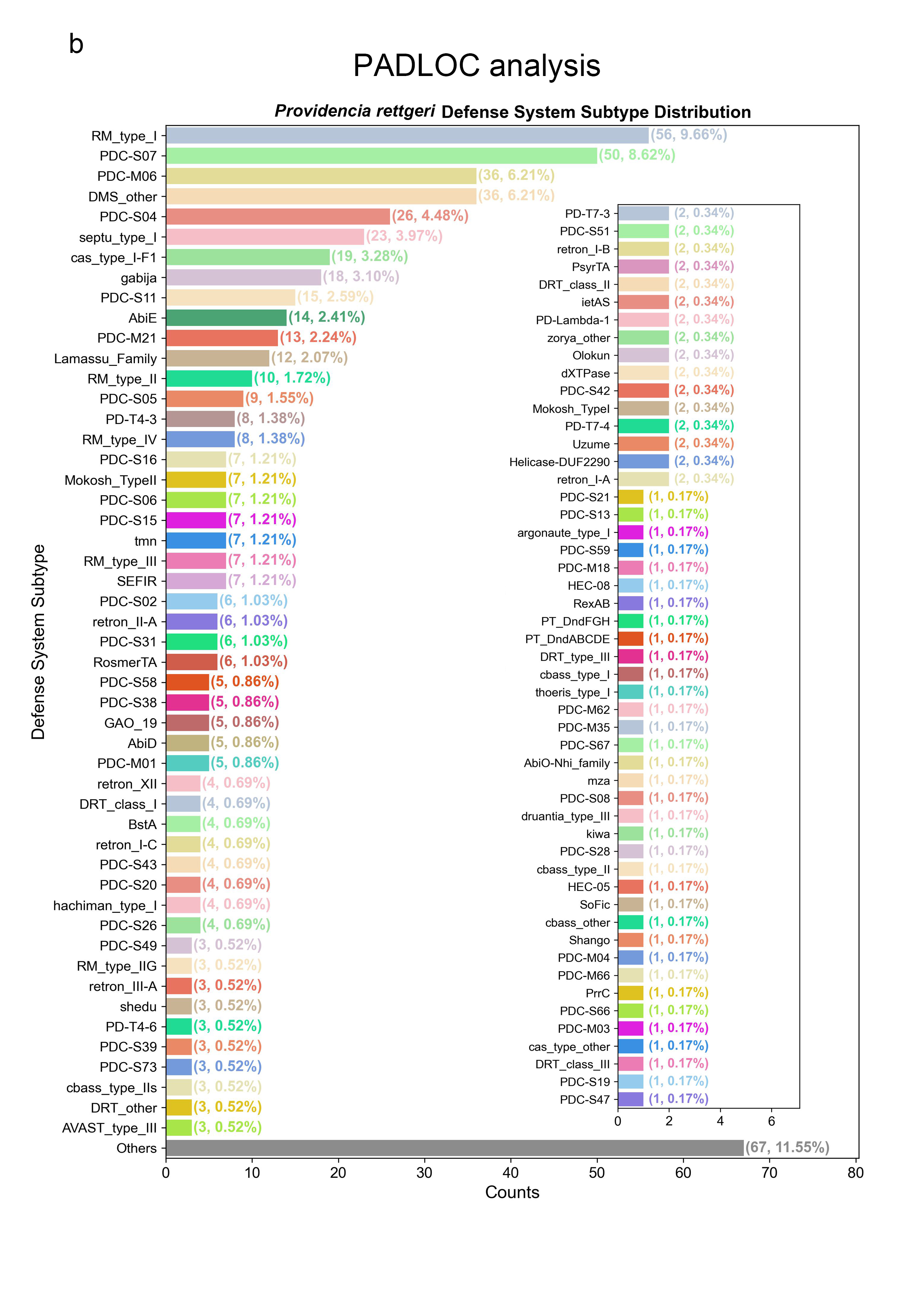

Supplement: Supplementary file 1 [file DataSheet1.zip › Supplementary_Figures/SuppFig3b.jpg]

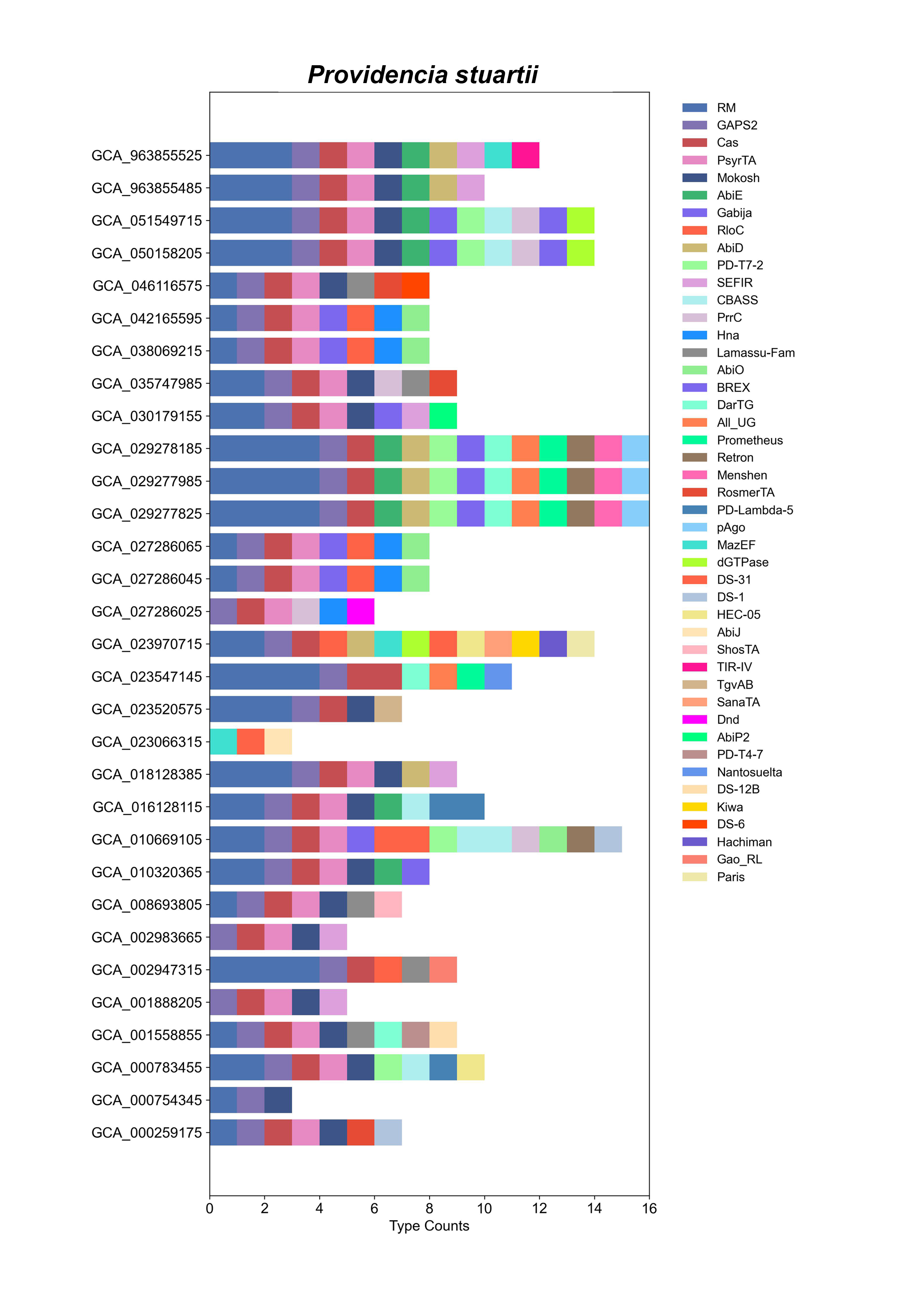

Supplement: Supplementary file 1 [file DataSheet1.zip › Supplementary_Figures/SuppFig4a.jpg]

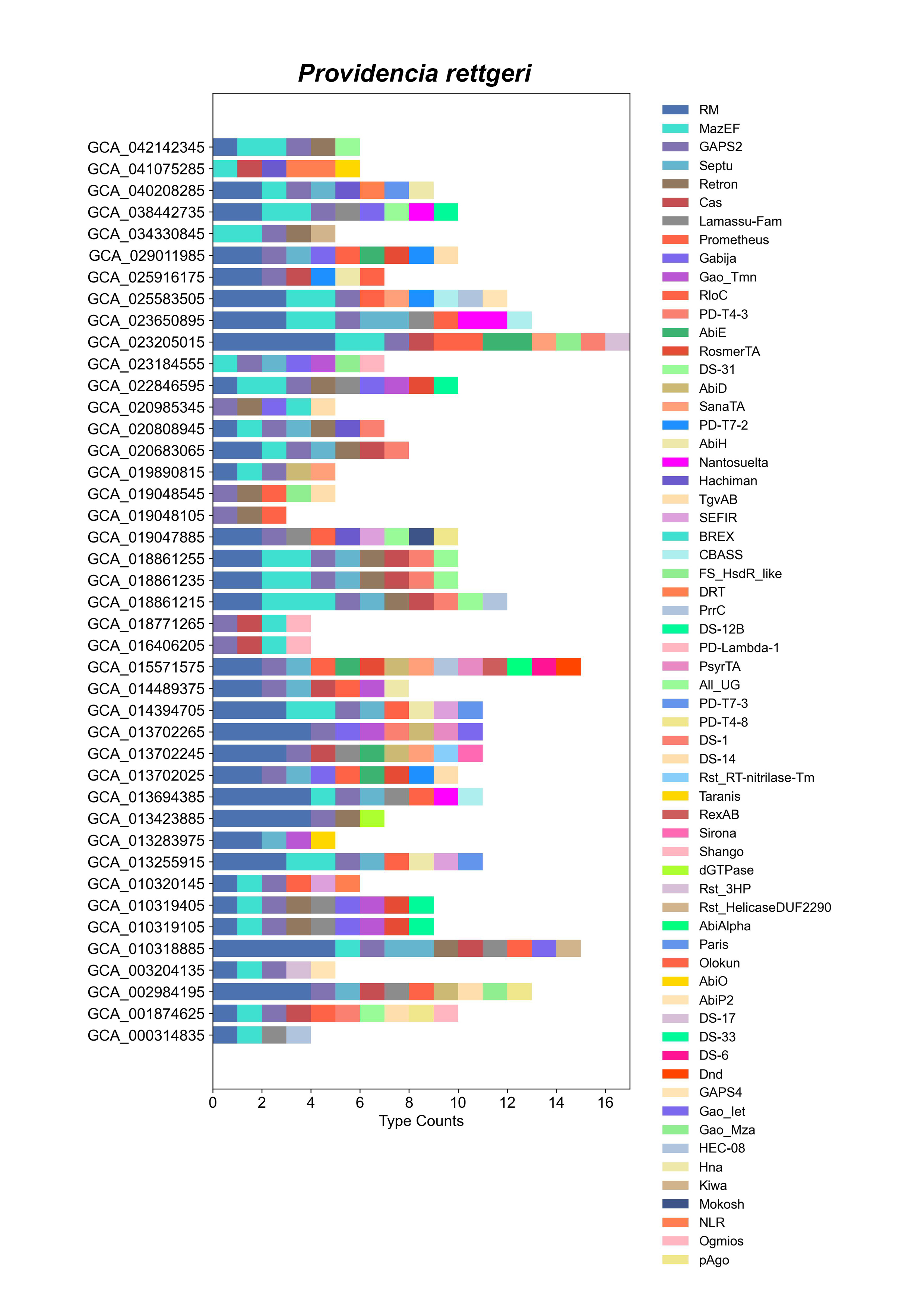

Supplement: Supplementary file 1 [file DataSheet1.zip › Supplementary_Figures/SuppFig4b.jpg]

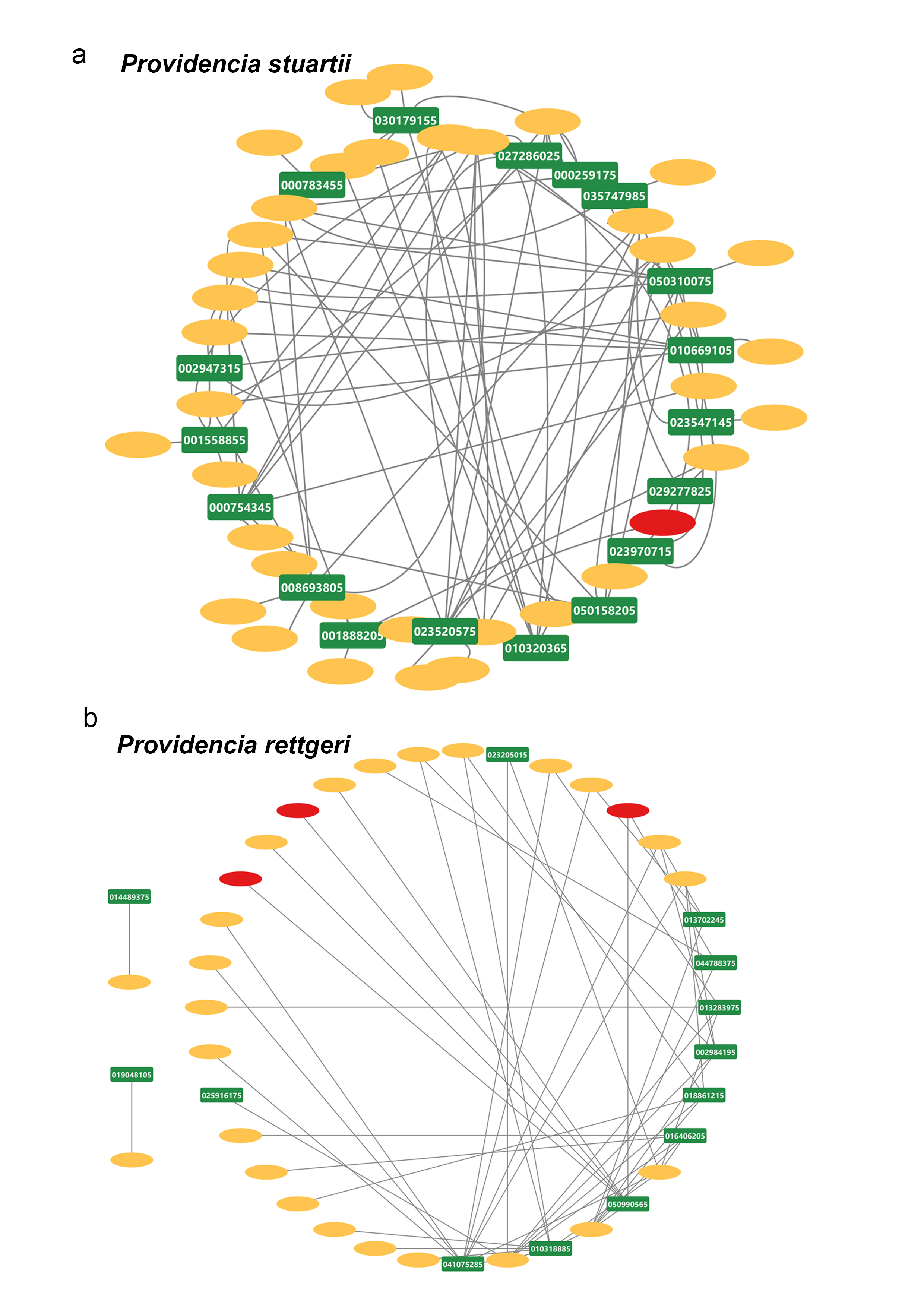

Supplement: Supplementary file 1 [file DataSheet1.zip › Supplementary_Figures/SuppFig5.jpg]

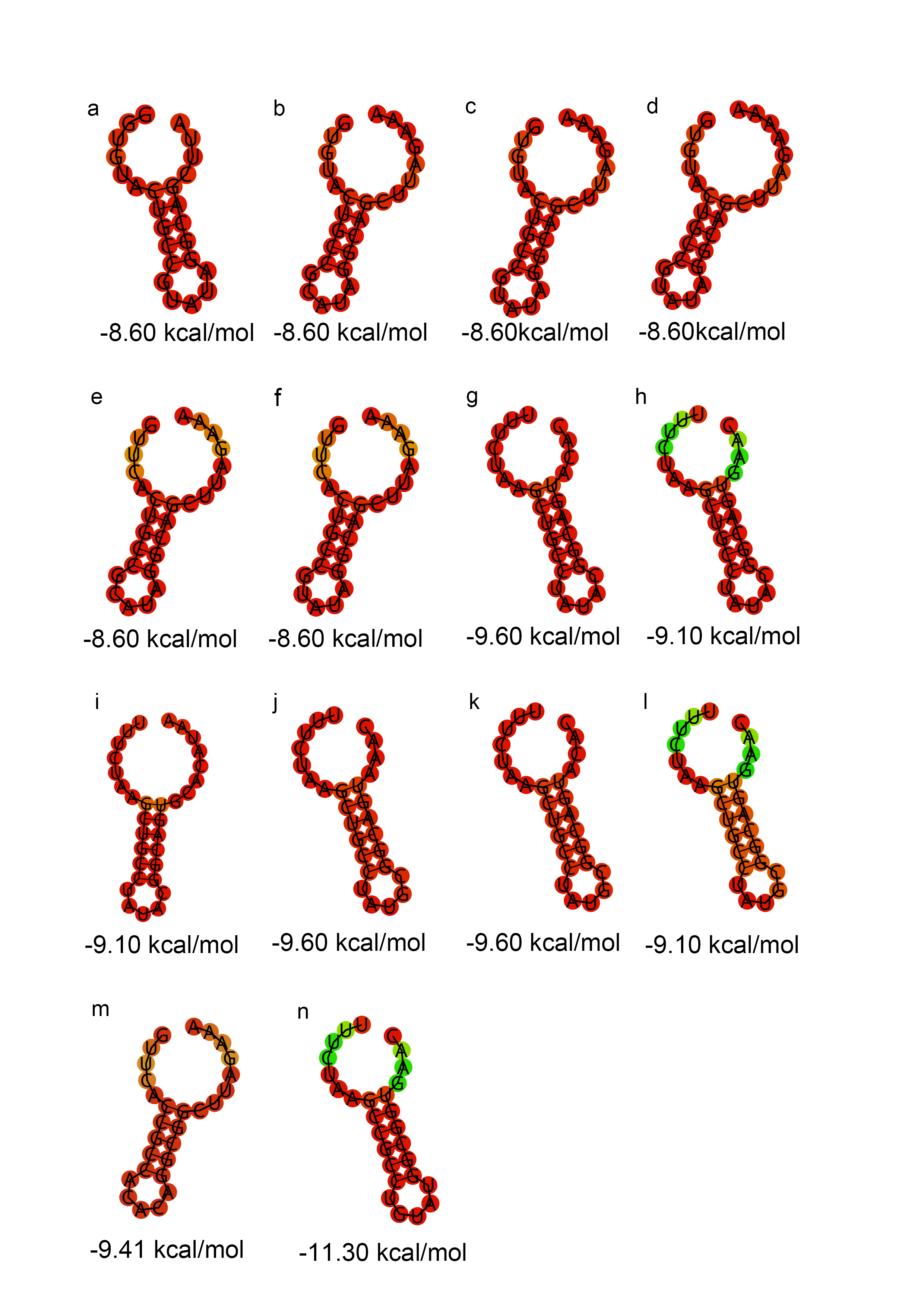

Supplement: Supplementary file 1 [file DataSheet1.zip › Supplementary_Figures/SuppFig6.jpg]

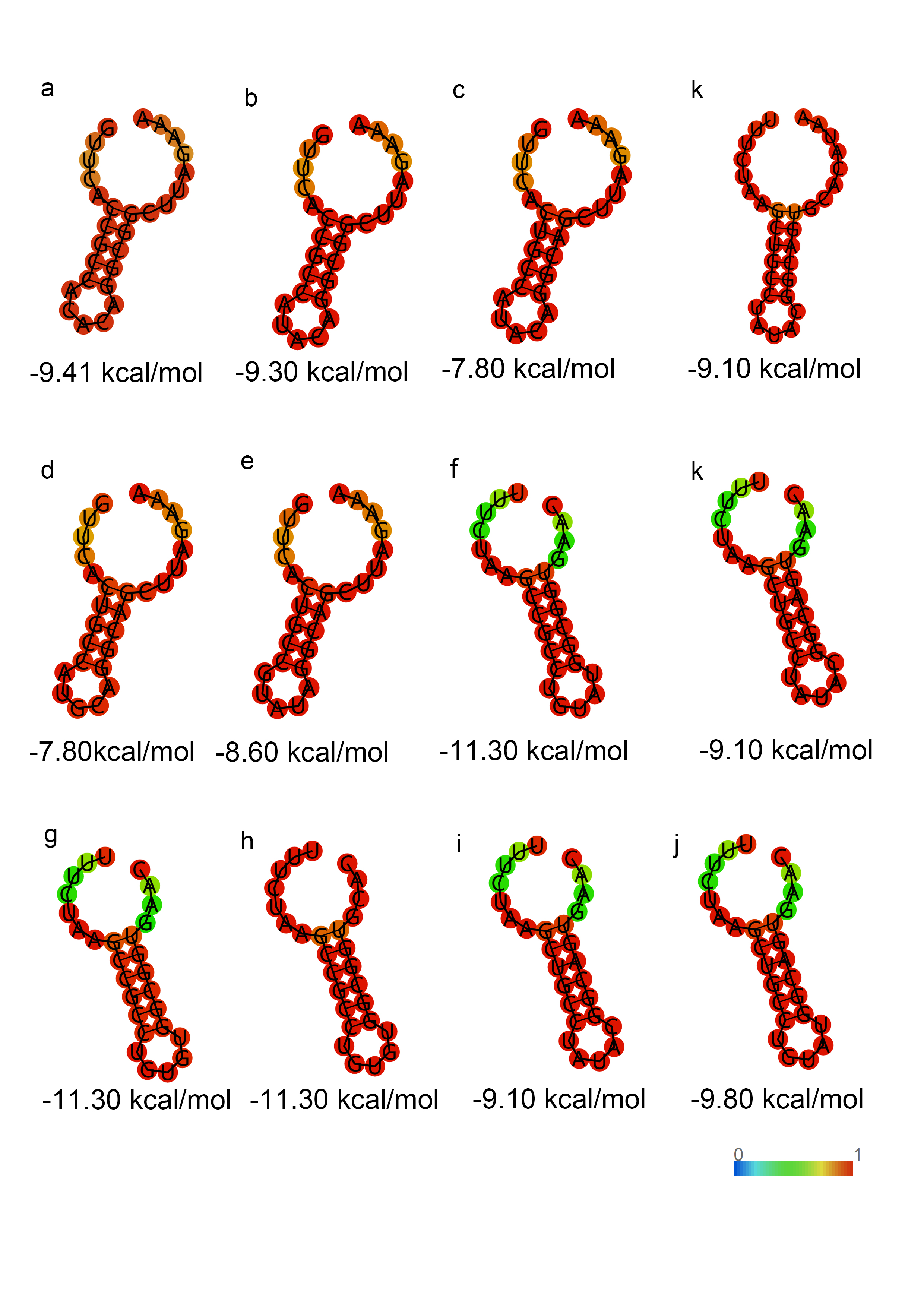

Supplement: Supplementary file 1 [file DataSheet1.zip › Supplementary_Figures/SuppFig7.jpg]
