# Supplementary material for: Characterization and diversity of defense systems in Providencia pathogen
Source: Front Immunol. 2026 Feb 12;17:1755933. doi: 10.3389/fimmu.2026.1755933 (PMC12935923; doi:10.3389/fimmu.2026.1755933)
Supplement: Supplementary file 2 [file DataSheet2.zip › Supplementary_Data/Supplemenatry_Figure_Legends.docx]

# Supplementary Figure 1

**Supplementary Figure 1.** Comparative Analysis of Immune Defense System Subtype Diversity in Providencia and Other Clinically Relevant Pathogens. (a) Defense system subtype abundance across bacterial genera associated with sepsis. This panel presents the number of distinct defense system subtypes (e.g., CRISPR-Cas type I-E, II-A; restriction-modification systems like R-M Type I, II, etc.) identified in major sepsis-associated bacterial genera. Unlike Figure 1, which counts defense types, this analysis includes specific subtypes within each system class. ****p* < 0.001 vs. Providencia; one-way ANOVA with Tukey’s post hoc test. (b) Species-level comparison of defense system subtype richness in *Providencia* and related pathogens. This panel extends the analysis to the species level, focusing on *P. stuartii* and *P. rettgeri*, along with key clinical isolates from other sepsis-causing species. Each colored dot represents an individual genome, with jitter applied to improve visualization of data distribution. Red crossbars denote the median (horizontal line) and interquartile range (vertical lines). The black * (*p* < 0.05) denote significance vs. P. stuartii, red * (*p* < 0.05) denote significance vs. P. rettgeri (one-way ANOVA with Tukey’s post hoc test).

# Supplementary Figure 2

**Supplementary Figure 2.** Defense system type distribution in Providencia contig and scaffold genomes. (a) P. stuartii (429 contig assemblies) defense system type distribution. Bar plot showing counts (and percentages) of defense system types in P. stuartii contig genomes. Dominant systems include RM (608, 5.31%) and Cas (429, 10.80%); the inset plot details the “Others” category (4.25% of systems). (b) P. stuartii (50 scaffold assemblies) defense system type distribution. Bar plot showing counts (and percentages) of defense system types in P. stuartii scaffold genomes. Dominant systems include RM (99, 18.44%) and Cas (51, 9.50%); the inset plot details the “Others” category (10.43% of systems). (c) P. rettgeri (334 contig assemblies) defense system type distribution. Bar plot showing counts (and percentages) of defense system types in P. rettgeri contig genomes. Dominant systems include RM (567, 8.50%) and GAPS2 (326, 10.64%); the inset plot details the “Others” category (6.04% of systems). (d) P. rettgeri (244 scaffold assemblies) defense system type distribution. Bar plot showing counts (and percentages) of defense system types in P. rettgeri scaffold genomes. Dominant systems include RM (548, 22.79%) and GAPS2 (241, 10.02%); the inset plot details the “Others” category (5.45% of systems).

# Supplementary Figure 3

**Supplementary Figure 3.** PADLOC-derived defense system subtype distribution in Providencia complete genomes. (a) P. stuartii (n = 31) defense system subtype distribution (PADLOC). Bar plot showing PADLOC-annotated defense system subtypes in P. stuartii complete genomes. Dominant subtypes include DMS_other (53, 10.43%) and cas_type_F-1 (30, 5.91%); the inset plot details the “Others” category (13.58% of subtypes). (b) P. rettgeri (n = 42) defense system subtype distribution (PADLOC). Bar plot showing PADLOC-annotated defense system subtypes in P. rettgeri complete genomes. Dominant subtypes include RM_type_I (56, 9.66%) and PDC-S07 (50, 8.62%); the inset plot details the “Others” category (11.55% of subtypes).

# Supplementary Figure 4

**Supplementary Figure 4.** Overview of Defense System Composition Across All Analyzed *P. stuartii* and *P. rettgeri* Strains. A complete inventory of defense system types across all *P. stuartii* (a) and *P. rettgeri* (b) strains included in this study, extending the analysis from Figure 4, which displayed only selected isolates. Each stacked bar represents an individual genome, with the horizontal axis indicating the total number of distinct defense system types present. The color scheme is consistent across panels, with each color corresponding to a specific defense system type as defined in the legend, enabling direct comparison across strains and species.

# Supplementary Figure 5

**Supplementary Figure 5.** Host-mobile genetic element (MGE) interaction networks of *P. stuartii* and *P. rettgeri.* (a) Host-MGE interaction network of *P. stuartii*. Green nodes represent bacterial host strains, with green boxes displaying the numerical portions of GenBank assembly accessions (GCA prefix omitted for space efficiency). Yellow ovals indicate phages, and red ovals denote plasmids. An edge connects a host and an MGE if the host’s CRISPR spacers match protospacers in the MGE sequence, filtered by >90% sequence identity, >80% query coverage, and e-value <0.001. (b) Host-MGE interaction network of *P. rettgeri*. Nodes, edges, and accession number labeling follow the same conventions as (a). All networks were constructed using the BacMGEnet computational pipeline, with non-redundant hosts and MGEs selected via a greedy algorithm, and visualized in Cytoscape.

# Supplementary Figure 6

**Supplementary Figure 6.** Analysis of RNA Secondary Structures and Minimum Free Energy (MFE) of CRISPR Repeat Sequences in All Analyzed Providencia stuartii Strains. Each panel (a–n) illustrates the minimum free energy (MFE) structure of a distinct CRISPR repeat locus, with nucleotides color-coded by base type (A: red, U: green, G: orange, C: blue) and stems and loops depicted to reflect thermodynamically stable folding patterns.

# Supplementary Figure 7

**Supplementary Figure 7.** Analysis of RNA Secondary Structures and Minimum Free Energy (MFE) of CRISPR Repeat Sequences in All Analyzed Providencia rettgeri Strains. Each panel (a–l) illustrates the minimum free energy (MFE) structure of a distinct CRISPR repeat locus, with nucleotides color-coded by base type (A: red, U: green, G: orange, C: blue) and stems and loops depicted to reflect thermodynamically stable folding patterns.
