# Supplementary material for: Characterization and diversity of defense systems in Providencia pathogen
Source: Front Immunol. 2026 Feb 12;17:1755933. doi: 10.3389/fimmu.2026.1755933 (PMC12935923; doi:10.3389/fimmu.2026.1755933)
Supplement: Supplementary file 2 [file DataSheet2.zip › Supplementary_Data/Supplemenatry_Tables.docx]

**Supplementary Table 1** The Accession IDs of the *Providencia* strains used in the study.

| Accession ID | Species |  | Accession_ID | Species |  |
| --- | --- | --- | --- | --- | --- |
| GCA_000259175 | *Providencia stuartii* |  | GCA_000314835 | *Providencia rettgeri* | |
| GCA_000754345 | *Providencia stuartii* |  | GCA_001874625 | *Providencia rettgeri* | |
| GCA_000783455 | *Providencia stuartii* |  | GCA_002984195 | *Providencia rettgeri* | |
| GCA_001558855 | *Providencia stuartii* |  | GCA_003204135 | *Providencia rettgeri* | |
| GCA_001888205 | *Providencia stuartii* |  | GCA_010318885 | *Providencia rettgeri* | |
| GCA_002947315 | *Providencia stuartii* |  | GCA_010319105 | *Providencia rettgeri* | |
| GCA_002983665 | *Providencia stuartii* |  | GCA_010319405 | *Providencia rettgeri* | |
| GCA_008693805 | *Providencia stuartii* |  | GCA_010320145 | *Providencia rettgeri* | |
| GCA_010320365 | *Providencia stuartii* |  | GCA_013255915 | *Providencia rettgeri* | |
| GCA_010669105 | *Providencia stuartii* |  | GCA_013283975 | *Providencia rettgeri* | |
| GCA_016128115 | *Providencia stuartii* |  | GCA_013423885 | *Providencia rettgeri* | |
| GCA_018128385 | *Providencia stuartii* |  | GCA_013694385 | *Providencia rettgeri* | |
| GCA_023066315 | *Providencia stuartii* |  | GCA_013702025 | *Providencia rettgeri* | |
| GCA_023520575 | *Providencia stuartii* |  | GCA_013702245 | *Providencia rettgeri* | |
| GCA_023547145 | *Providencia stuartii* |  | GCA_013702265 | *Providencia rettgeri* | |
| GCA_023970715 | *Providencia stuartii* |  | GCA_014394705 | *Providencia rettgeri* | |
| GCA_027286025 | *Providencia stuartii* |  | GCA_014489375 | *Providencia rettgeri* | |
| GCA_027286045 | *Providencia stuartii* |  | GCA_015571575 | *Providencia rettgeri* | |
| GCA_027286065 | *Providencia stuartii* |  | GCA_016406205 | *Providencia rettgeri* | |
| GCA_029277825 | *Providencia stuartii* |  | GCA_018771265 | *Providencia rettgeri* | |
| GCA_029277985 | *Providencia stuartii* |  | GCA_018861215 | *Providencia rettgeri* | |
| GCA_029278185 | *Providencia stuartii* |  | GCA_018861235 | *Providencia rettgeri* | |
| GCA_030179155 | *Providencia stuartii* |  | GCA_018861255 | *Providencia rettgeri* | |
| GCA_035747985 | *Providencia stuartii* |  | GCA_019047885 | *Providencia rettgeri* | |
| GCA_038069215 | *Providencia stuartii* |  | GCA_019048105 | *Providencia rettgeri* | |
|  |  |  | GCA_019048545 | *Providencia rettgeri* | |
|  |  |  | GCA_019890815 | *Providencia rettgeri* | |
|  |  |  | GCA_020683065 | *Providencia rettgeri* | |
|  |  |  | GCA_020808945 | *Providencia rettgeri* | |
|  |  |  | GCA_020985345 | *Providencia rettgeri* | |
|  |  |  | GCA_022846595 | *Providencia rettgeri* | |
|  |  |  | GCA_023184555 | *Providencia rettgeri* | |
|  |  |  | GCA_023205015 | *Providencia rettgeri* | |
|  |  |  | GCA_023650895 | *Providencia rettgeri* | |
|  |  |  | GCA_025583505 | *Providencia rettgeri* | |
|  |  |  | GCA_025916175 | *Providencia rettgeri* | |
|  |  |  | GCA_029011985 | *Providencia rettgeri* | |
|  |  |  | GCA_034330845 | *Providencia rettgeri* | |
|  |  |  | GCA_038442735 | *Providencia rettgeri* | |
|  |  |  | GCA_040208285 | *Providencia rettgeri* | |
|  |  |  | GCA_041075285 | *Providencia rettgeri* | |
|  |  |  | GCA_042142345 | *Providencia rettgeri* | |
